# PGE_2_ alters chromatin through H2A.Z-variant enhancer nucleosome modification to promote hematopoietic stem cell fate

**DOI:** 10.1073/pnas.2220613120

**Published:** 2023-05-01

**Authors:** Audrey Sporrij, Avik Choudhuri, Meera Prasad, Brejnev Muhire, Eva M. Fast, Margot E. Manning, Jodi D. Weiss, Michelle Koh, Song Yang, Robert E. Kingston, Michael Y. Tolstorukov, Hans Clevers, Leonard I. Zon

**Affiliations:** ^a^Department of Stem Cell and Regenerative Biology, Harvard University, Cambridge, MA 02138; ^b^Stem Cell Program and Division of Hematology/Oncology, Boston Children’s Hospital, Boston, MA 02115; ^c^Department of Molecular Biology, Massachusetts General Hospital and Harvard Medical School, Boston, MA 02114; ^d^Department of Informatics and Analytics, Dana-Farber Cancer Institute, Boston, MA 02215; ^e^Oncode Institute, Hubrecht Institute, Royal Netherlands Academy of Arts and Sciences and University Medical Center Utrecht, Utrecht 3584 CT, The Netherlands; ^f^Princess Máxima Center for Pediatric Oncology, Utrecht 3584 CS, The Netherlands; ^g^HHMI, Harvard Stem Cell Institute, Boston, MA 02115; ^h^Harvard Medical School, Harvard Stem Cell Institute, Boston, MA 02115

**Keywords:** hematopoietic stem cell, prostaglandin, chromatin

## Abstract

Prostaglandin E2 (PGE_2_) and 16,16-dimethyl-PGE_2_ (dmPGE_2_) are important regulators of hematopoietic stem and progenitor cell (HSPC) fate and offer potential to enhance stem cell therapies [C. Cutler *et al.* *Blood* **122**, 3074–3081(2013); W. Goessling *et al.* *Cell Stem Cell* **8**, 445–458 (2011); W. Goessling *et al.* *Cell* **136**, 1136–1147 (2009)]. Here, we report that PGE_2_-induced changes in chromatin at enhancer regions through histone-variant H2A.Z permit acute inflammatory gene induction to promote HSPC fate. We found that dmPGE_2_-inducible enhancers retain MNase-accessible, H2A.Z-variant nucleosomes permissive of CREB transcription factor (TF) binding. CREB binding to enhancer nucleosomes following dmPGE_2_ stimulation is concomitant with deposition of histone acetyltransferases p300 and Tip60 on chromatin. Subsequent H2A.Z acetylation improves chromatin accessibility at stimuli-responsive enhancers. Our findings support a model where histone-variant nucleosomes retained within inducible enhancers facilitate TF binding. Histone-variant acetylation by TF-associated nucleosome remodelers creates the accessible nucleosome landscape required for immediate enhancer activation and gene induction. Our work provides a mechanism through which inflammatory mediators, such as dmPGE_2_, lead to acute transcriptional changes and modify HSPC behavior to improve stem cell transplantation.

## Significance

Hematopoietic stem and progenitor cells (HSPCs) require tight transcriptional regulation. In-depth knowledge on how epigenetic mechanisms regulate chromatin landscape in order to control gene expression, particularly in response to external stimuli, will be key to understand the pathogenesis of hematologic disorders and improve blood stem cell-based therapies. Prostaglandin E2 (PGE_2_) and its metabolically resistant analog 16,16-dimethyl-PGE2 (dmPGE_2_) were previously identified as a potent external stimulus to enhance HSPC engraftment. PGE_2_ has been evaluated in various clinical trials to improve engraftment of stem cells. We identified that dmPGE_2_ mediates chromatin flexibility at HSPC-specific enhancers through histone-variant H2A.Z acetylation to promote master transcription factor (TF) binding and reinforce expression of genes involved in stem cell fate and engraftment.

Hematopoietic stem cells are characterized by their unique ability to self-renew and differentiate into all mature blood cell lineages. During normal homeostasis and in conditions of stress such as injury or inflammation, HSPCs maintain an appropriate balance of the hematopoietic system. Hematopoietic stem and progenitor cells (HSPCs) sense and respond to a variety of extrinsic signals that regulate their quiescence, proliferation, and differentiation (1–3).

Prostaglandins are physiologically active lipids produced in response to mechanical, chemical, or immunological stimuli. They sustain a variety of homeostatic and pathogenic functions. This includes roles in the inflammatory response. Prostaglandin E2 (PGE_2_) is one of the most abundant prostaglandins produced in the body (4). PGE_2_ and its stable derivative dmPGE_2_ act as important regulators of vertebrate HSPC development and homeostasis (1, 5). We previously demonstrated that ex vivo pulse exposure of HSPCs to dmPGE_2_ enhances engraftment and self-renewal in mice and clinical studies indicate benefits for hematopoietic stem cell transplantation (HSCT) outcomes in humans (6, 7). DmPGE_2_ predominantly exerts its effects by binding to the PGE_2_ receptor (EP) subtypes EP2 and EP4 on HSPCs^2^. Interaction with these G-coupled protein receptors enhances intracellular cAMP levels, which activates signaling cascades and downstream effectors, for instance Wnt and β-catenin (8). Improved engraftment presumably results from upregulation of genes implicated in HSPC homing, such as *CXCR4* (9). As enhancement of HSPC function by external stimuli supports a strategy to improve HSCTs, understanding the mechanism of gene regulation in response to inductive signals can provide a significant clinical opportunity.

A main mechanism of adaptation involves the activation of TFs that are downstream of signal transduction pathways to ensure appropriate transcriptional responses upon stimulation (10–12). In higher eukaryotes, gene expression is regulated by the coordinated action of enhancers and promoters (13). Stimuli-responsive TFs (STFs) tend to operate within the cis-regulatory repertoire that is established during cell fate specification and maintained by constitutive binding of lineage-specific master TFs (MTFs) (14). The access of STFs to these regulatory elements and their interaction with cofactors, such as transcriptional activators and chromatin remodeling complexes, depend largely on the local chromatin architecture (15). Generally accepted features of active regulatory regions include open chromatin conformation, histone modifications, and TF binding (16). While promoters consist of a nucleosome-depleted region that is established by chromatin remodelers, general TFs, and the basal transcription machinery, the typical chromatin organization and nucleosome configuration at enhancers remain unclear (17).

Here, we sought to address how transcriptional induction is regulated during the HSPC response to dmPGE_2_. We exploited inducible TF binding to chromatin and identified, and then mechanistically dissected, enhancers controlling inflammatory gene expression changes in HSPCs. We assessed the chromatin accessibility and nucleosome organization of regulatory regions responsive to dmPGE_2_ and studied how the higher-order chromatin structures changed following induction. We found that stimuli-induced enhancers retained MNase-accessible nucleosomes. These enhancer nucleosomes were enriched with the noncanonical histone-variant H2A.Z and remodeled but not evicted during acute stimulation. Rather than prohibiting TF binding, we observed enrichment of the dmPGE_2_-responsive TF CREB at accessible nucleosomes within inducible enhancers. CREB binding is concomitant with deposition of the chromatin remodelers p300 and Tip60 that acetylate histone-variant H2A.Z following dmPGE_2_ stimulation. This may further improve nucleosome accessibility at stimuli-responsive enhancers and allow for binding of additional TFs and coactivator complexes. We show that the nucleosome organization at enhancers is not exclusively repressive to gene regulation but favors STF binding, which enables rapid enhancer activation and inflammatory gene induction. Our study uncovers a mechanism at the chromatin level that supports acute changes in gene expression, underlying the biologic effect of PGE_2_ to induce changes in stem cell engraftment potential.

## Results

### CREB Regulates Gene Expression Changes through Binding at Enhancer Elements.

**Fig. 1.**
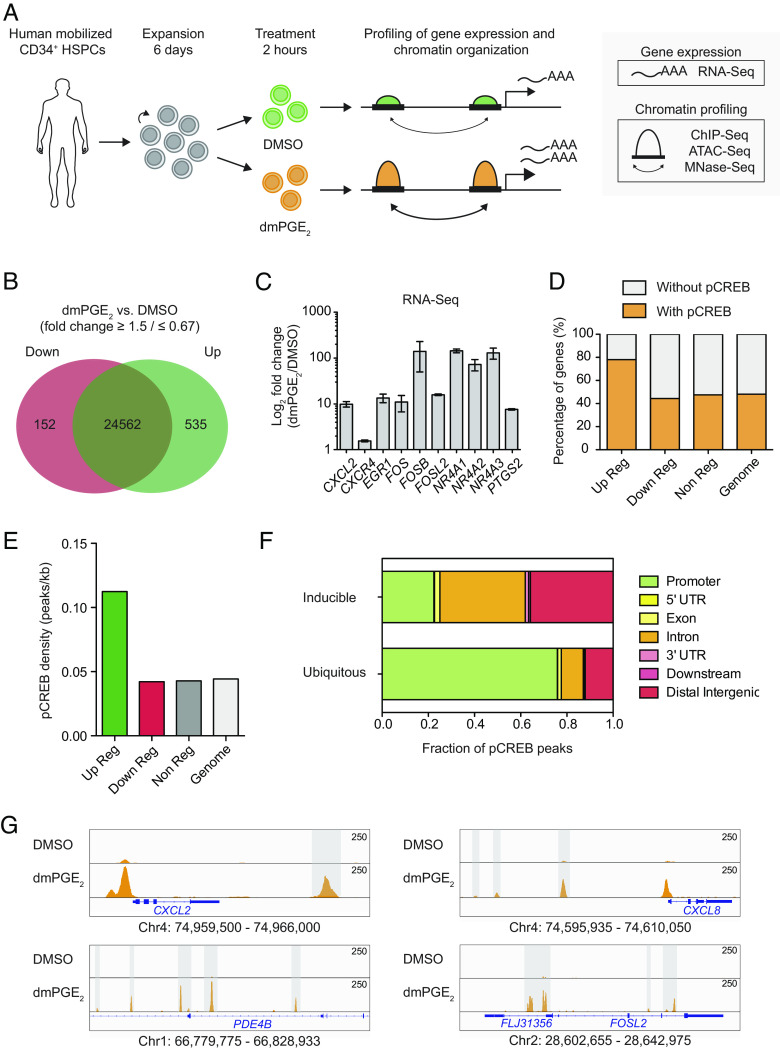
Phospho-CREB regulates dmPGE_2_-induced gene expression changes through binding at distal regulatory elements. (*A*) Schematic representation of the experimental approach used in this study. Cells are stimulated for 2 h with dmPGE_2_ or vehicle control (DMSO) after which transcriptome and epigenome profiling was performed. (*B*) Venn diagram showing the number of genes up-regulated in green (535) and down-regulated in red (152) in CD34^+^ HSPCs after 2 h of dmPGE_2_ stimulation in comparison to control-treated cells, as determined by RNA-Seq analysis. DEG criteria: FPKM ≥1 after treatment; fold change ≥1.5 or ≤0.67 (n = 3 biologically independent experiments). (*C*) Examples of genes identified as differentially expressed by RNA-Seq (n = 3 biologically independent experiments; mean values ± SEM). (*D*) Number of genes containing at least one pCREB peak in the proximity after dmPGE_2_ stimulation. pCREB peaks assigned to a gene when located within a window from −5 kb upstream of the (TSS) to +5 kb downstream of the TTS were considered (n = 2 biologically independent ChIP-Seq experiments). (*E*) Correlation between pCREB binding and gene expression in response to dmPGE_2_. pCREB density was calculated by dividing the total number of pCREB peaks associated to each gene category (up−, down−, and nonregulated genes) by the total amount of base pairs that this category occupies in the genome. pCREB peaks were assigned to a gene when located from +5 kb upstream of the TSS to +5 kb downstream of the TTS. Peak density in the genome was calculated by considering random distribution of pCREB sites in the whole genome. (*F*) Genomic distribution of unique pCREB peaks (inducible; present only after dmPGE_2_ stimulation) versus ubiquitous pCREB peaks. (*G*) Enrichment of pCREB binding at four representative dmPGE_2_ response genes: *CXCL2* (promoter, intergenic), *CXCL8* (promoter, intergenic), *PDE4B* (intronic), and *FOSL2* (promoter, intronic). Gray bars indicate intronic and intergenic pCREB peaks. Genomic location of presented window is indicated at the bottom of the panels.

We sought to define the molecular mechanisms that underlie the transcriptional response of HSPCs to dmPGE_2_. dmPGE_2_ is a synthetic stable derivative of PGE_2_. While this metabolically stable PGE_2_ analog is not naturally generated in vivo, it has previously been shown to act as an important regulator of vertebrate HSPC development in ways similar to PGE_2_ (1, 8). We exposed human mobilized peripheral blood CD34^+^ HSPCs to 10μM dmPGE_2_ for 2 h and performed extensive gene expression and chromatin profiling (Fig. 1*A*). Using RNA-sequencing (RNA-Seq), we identified a total of 687 consistent differentially expressed genes (DEGs) after 2 h of dmPGE_2_ treatment, when compared to vehicle (DMSO)-treated control cells (Fig. 1*B*). The effect of dmPGE_2_ on gene expression was overwhelmingly stimulatory. More specifically, 535 genes were ≥1.5-fold up-regulated. This includes a significant number of genes involved in cell migration and cell cycle regulation (*SI Appendix*, Fig. S1 *A* and *C*). Among the up-regulated set were genes representative of dmPGE_2_/cAMP/PKA signaling, including *PDE4B* and *PTGS2* (18); several chemokines and cytokines, such as *CXCL2* and *CXCL8* (19); and genes known to restrict HSPC proliferation and differentiation, for instance *NR4A1* and *JUNB* (20, 21) (Fig. 1*C* and *SI Appendix*, Table S1). We validated the gene expression signature observed by RNA-Seq through RT-qPCR in HSPCs for representative genes (*SI Appendix*, Fig. S1*D*). A total of 152 genes showed a ≤0.67-fold decrease in expression (*SI Appendix*, Fig. S1*B* and  Table S1). Among the repressed genes, we found enrichment of genes that regulate cell division, such as *HOXB4* and *CCNF* (22). To evaluate the functional impact of gene expression changes in human HSPCs, we assessed cell migration after dmPGE_2_ treatment in vitro. By transwell migration assay, which served as a proxy for the previously observed engraftment phenotype (1), we observed greater migration of HSPCs after exposure to dmPGE_2_ (*SI Appendix*, Fig. S1*E*). These data showed that enhanced engraftment in vivo after dmPGE_2_ stimulation is, in part, driven by transcriptional induction of migration genes.

The TF CREB has previously been associated with dmPGE_2_ signaling (23). Indeed, our transcriptomic analysis revealed that 30% (158/535) of up-regulated genes are known targets of CREB (*SI Appendix*, Fig. S2*A*). CREB binds to cyclic-AMP response elements (CREs) near its target genes where it, upon phosphorylation at serine 133 (S133) by protein kinases, promotes the recruitment of coactivator proteins (23). This increases transcription of CREB-dependent genes (24). One of the protein kinases that phosphorylates CREB at S133 is protein kinase A (PKA). We assessed S133 phosphorylation of CREB in HSPCs after dmPGE_2_ treatment. Western blot analysis revealed increased abundance of S133 phosphorylated CREB (pCREB), while total TF protein levels remain unaltered (*SI Appendix*, Fig. S2*B*). To correlate pCREB with gene induction, we performed chromatin immunoprecipitation sequencing (ChIP-Seq) with an antibody against pCREB. We found 31,198 binding sites (False Discovery Rate < 0.1%) in CD34^+^ HSPCs treated for 2 h with dmPGE_2_ compared to 8,332 sites in DMSO (*SI Appendix*, Fig. S2*C* and Table S2). Correlation of pCREB occupancy to DEGs showed enrichment of pCREB at genes induced by dmPGE_2_ (Fig. 1*D*). In fact, 79% of the up-regulated genes contained at least one pCREB peak in the proximity, that is a window from −5 kb upstream of the transcription start site (TSS) to +5 kb downstream of the TTS, after dmPGE_2_ stimulation compared to 47% prior to treatment (Fig. 1*D*). This value increased to 85% by extending the window up to −100 kb from the TSS to +25 kb from TTS (*SI Appendix*, Fig. S2*C*). Besides a higher percentage of up-regulated genes containing pCREB, the TF density was also >2 times higher at up-regulated genes compared to noninduced genes (Fig. 1*E* and *SI Appendix*, Fig. S2*D*). We observed no clear correlation between the magnitude of the transcriptional response and the density of pCREB (*SI Appendix*, Fig. S2*F*). Genes down-regulated by dmPGE_2_ showed no additional enrichment in pCREB compared to noninduced genes. Since both the phosphorylation status and TF occupancy of CREB may change in response to dmPGE_2_, we performed ChIP-Seq using a CREB antibody that is nondiscriminating against its phosphorylation status and compared the total CREB occupancy against pCREB-specific occupancy across the genome. These data demonstrated that, relative to total CREB levels, dmPGE_2_ preferentially induced pCREB binding near dmPGE_2_-responsive genes (*SI Appendix*, Fig. S2*G*). Overall, these data suggested that dmPGE_2_ regulates pCREB activity and genomic binding near transcriptionally induced genes.

We next assessed the genomic location of pCREB-bound regions. A total of 23,386 (75%) sites displayed unique pCREB enrichment (“inducible” pCREB regions) in dmPGE_2_-treated HSPCs compared to control-treated cells. The other 7,812 (25%) pCREB sites were present in both control and stimulated HSPCs (“ubiquitous” pCREB regions). The majority of inducible pCREB sites were located distal to the TSS of genes, with a strong representation in intronic and intergenic sequences (Fig. 1 *F* and *G*). Ubiquitous pCREB sites were predominantly enriched in promoter regions. This indicated that pCREB binding at putative distal regulatory elements plays a pivotal role in up-regulating dmPGE_2_ response genes.

### Inducible Enhancers Gain Master and Signaling TF Binding.

**Fig. 2.**
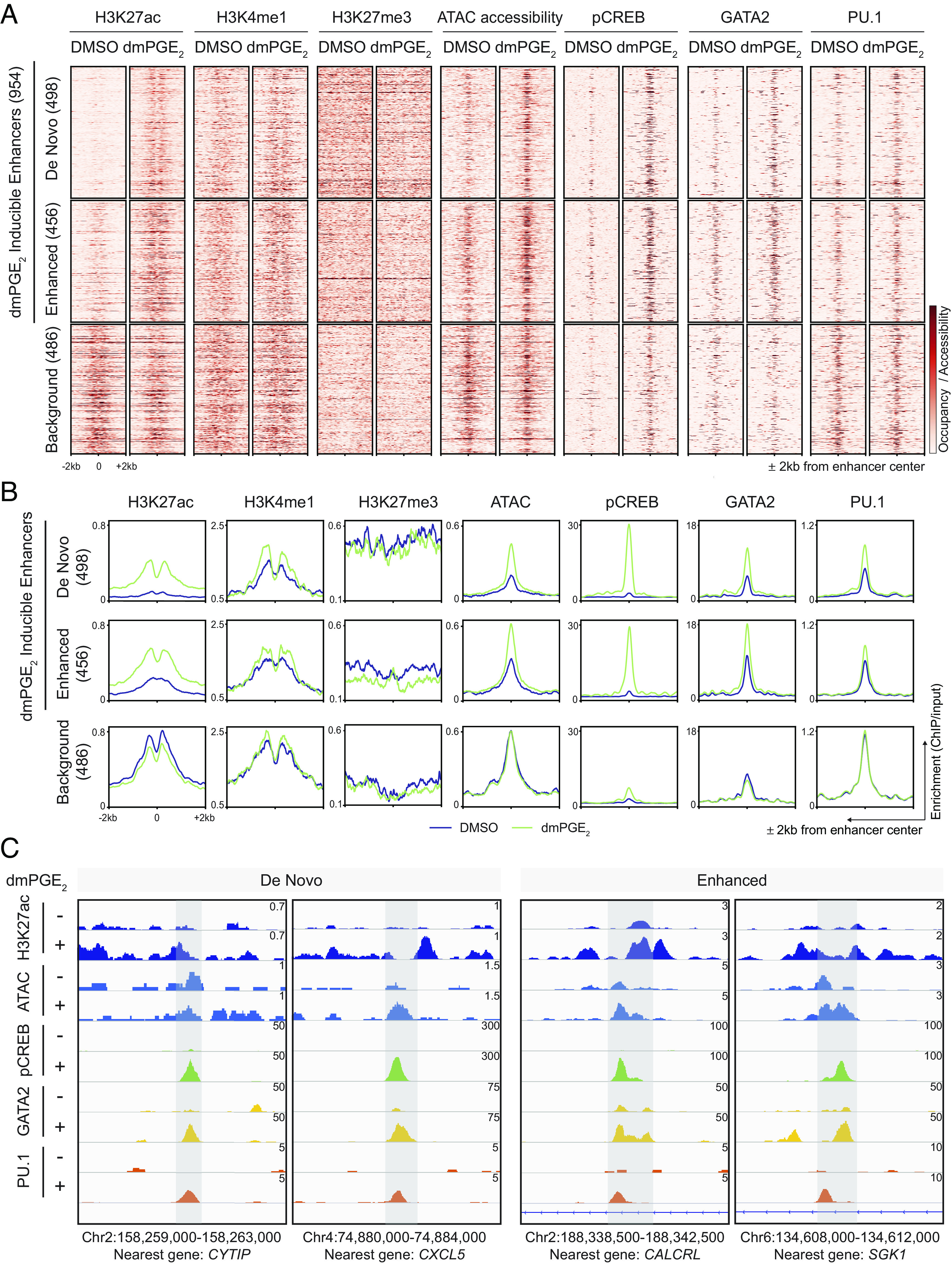
Stimuli-responsive enhancers gain chromatin accessibility and TF binding after dmPGE_2_ stimulation. (*A* and *B*) Heat maps (*A*) and average enrichment profiles (*B*) of histone marks, ATAC accessibility, and TFs at enhancers before and after dmPGE_2_ treatment. H3K27ac-enriched regions identified by ChIP-Seq are classified as de novo, enhanced, or background enhancers according to the change in H3K27ac levels observed following dmPGE_2_ stimulation (n = 2 biologically independent experiments). A randomly sampled, comparable number of background enhancers (486) is shown. (*C*) Enrichment of histone mark, ATAC accessibility, and TF binding in response to dmPGE_2_ at four representative stimuli-response enhancers. Genomic location of presented window and nearest gene is indicated at the *bottom* of the panel.

To understand the epigenetic impact of dmPGE_2_ on distal regulatory elements, we determined the effects of a 2-h stimulation on the state of enhancers in HSPCs. H3K27ac distinguishes active enhancers from primed and poised enhancer elements that are marked by H3K4me1 alone or with H3K27me3, respectively (25). We performed ChIP-Seq for these histone modifications. We assessed H3K27ac enrichment in control and dmPGE_2_-stimulated HSPCs and identified a total of 25,998 active distal regulatory elements (*SI Appendix*, Table S3). Enhancers that regulate stimulus-responsive gene programs can be distinguished from other active enhancers by their specific increase in H3K27ac upon receipt of the stimulus (26–28). Comparison of H3K27ac enrichment between control and dmPGE_2_-treated HSPCs allowed us to identify putative distal enhancers involved in the response to dmPGE_2_. We identified a total of 954 (3.7%) enhancers that gained significant enrichment in H3K27ac following dmPGE_2_ treatment (*SI Appendix*, Fig. S3 *A* and *B* and *Materials and Methods*). A subset of these stimuli-inducible enhancers (498/954, 52%) were only detected as active regulatory regions after dmPGE_2_ stimulation. While these “de novo” enhancers were depleted of H3K27ac prior to stimulation, their H3K4me1^high^/H3K27me3^low^ state indicated that de novo enhancers reside in a primed state prior to activation (Fig. 2 *A* and *B*). The other fraction of dmPGE_2_-inducible enhancers (456/954, 48%) displayed significant enhancement in H3K27ac enrichment after dmPGE_2_ treatment (“enhanced”, Fig. 2*A*, and *SI Appendix*, Fig. S3*C*). Since the number of noninducible enhancers (25,044 regions, 96.3%) is much larger than the set of inducible enhancers, we randomly sampled a comparable number of noninducible enhancers (“background”, 486) to ensure that observed differences are not due to variations in the size of defined enhancer categories. To assess epigenetic transitions at inducible enhancers, we profiled genome accessibility by assay for transposase-accessible chromatin sequencing (ATAC-Seq). We found a profound increase in DNA accessibility at inducible enhancers compared to background enhancers after dmPGE_2_ stimulation (Fig. 2 *A* and *B*), which is a sign of active chromatin reorganization (29). In addition, the mean ATAC-Seq signal prior to activation of de novo enhancers suggested preexisting, yet minimal, accessibility before stimulation. This revealed that dmPGE_2_ stimulation results in rapid activation of stimuli-responsive enhancers that is concomitant with increased chromatin accessibility.

It is well known that TFs act as anchors to recruit chromatin remodelers to regulate gene expression (12). Most STFs do not possess pioneering activity and therefore preferentially bind DNA elements located within nucleosome-depleted regions. Moreover, STFs often land at regulatory elements predefined by lineage-specific MTF that have pioneer functions (27, 30). Given these insights, we assessed both pCREB occupancy and binding of the HSPC MTFs GATA2 and PU.1 at enhancers prior to and after dmPGE_2_ treatment. We found that pCREB colocalized with GATA2 and PU.1 at stimuli-responsive enhancers (Fig. 2 *A* and *C*). We moreover observed that inducible enhancers not only gain pCREB but also GATA2 and PU.1 enrichment after dmPGE_2_ stimulation (Fig. 2 *B* and *C*). No enrichment in MTF occupancy was observed in background enhancers (Fig. 2 *A* and *B* and *SI Appendix*, Fig. S3*E*). These data showed that dmPGE_2_-inducible enhancers gain both STF and additional MTF deposition following stimulation.

**Fig. 3.**
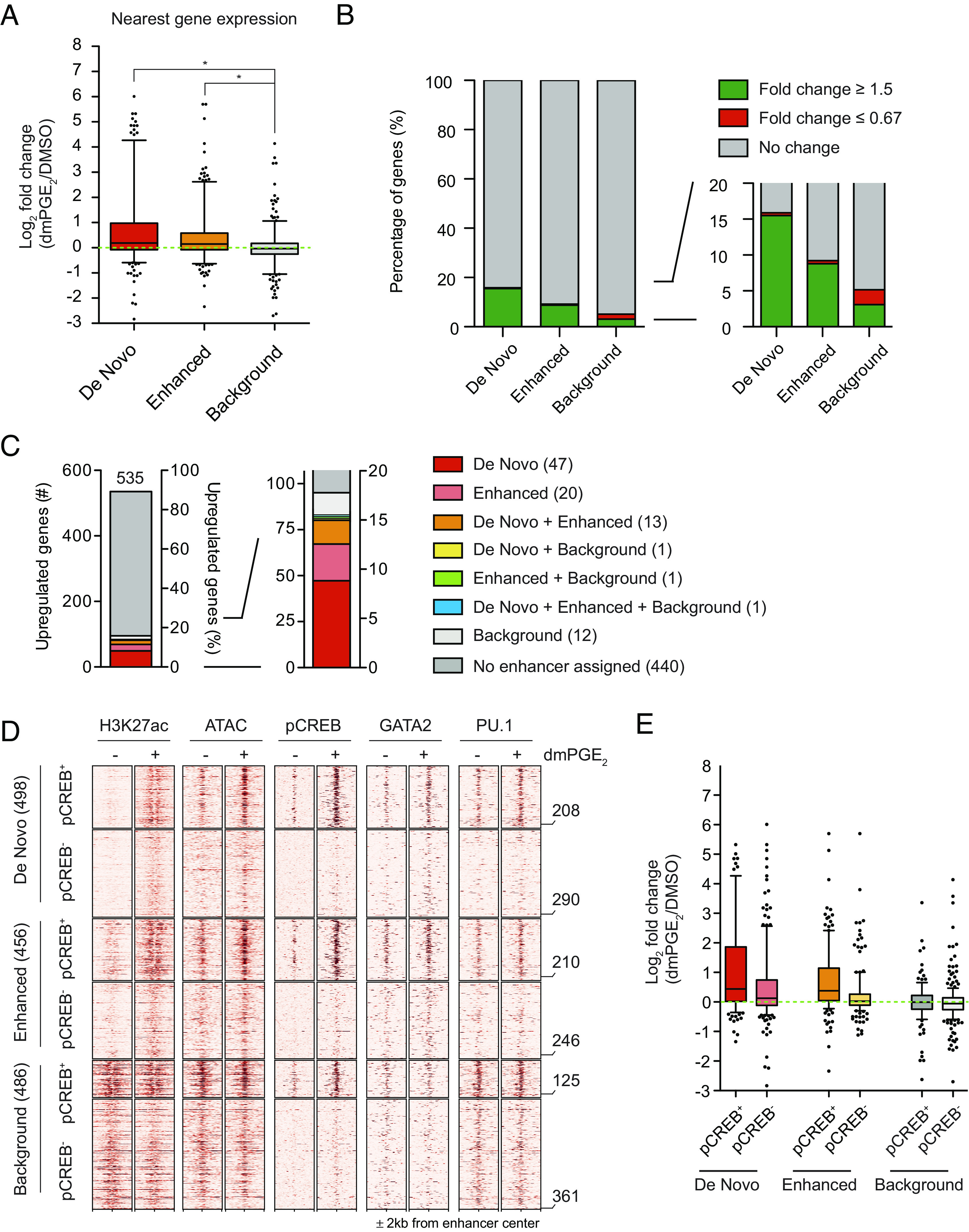
Stimuli-responsive enhancers mediate dmPGE_2_-induced gene expression changes. (*A*) Gene expression changes of genes associated with stimuli-responsive and background enhancers. Enhancers were assigned to an individual nearest gene. Only genes with a mapped TSS within 15 kb of an enhancer were considered. Box plots shows median, 25th and 75th percentiles, whiskers are from 5th and 95th percentiles. Dots indicate outliers. (*B*) Percentages of enhancer nearest genes with fold changes in expression ≥1.5-fold or ≤0.67-fold for each enhancer category. (*C*) Up-regulated genes with a fold change in expression ≥1.5 (535) and their associated enhancers. (*D*) Heat maps of H3K27ac, ATAC accessibility, and TFs around enhancers before and after dmPGE_2_ treatment. De novo, enhanced, or background enhancers were subset based on the presence or absence of pCREB after dmPGE_2_. A randomly sampled, comparable number of background enhancers (486) is shown. Numbers of enhancers within each subset is indicated on the right of the heatmap. (*E*) Gene expression changes of genes associated with pCREB-positive and pCREB-negative enhancers. Enhancers were assigned to an individual nearest gene. Only genes with a mapped TSS within 15 kb of an enhancer were considered. Box plots shows median, 25th and 75th percentiles, whiskers are from 10th and 90th percentiles. Dots indicate outliers. For all analyses presented here, a randomly sampled set of background enhancers (486) was used.

The contribution of distal regulatory regions for transcriptional activation of dmPGE_2_ target genes is illustrated by the changes in the expression of genes regulated by stimuli-responsive enhancers compared to those regulated by background enhancers (Fig. 3*A* and *SI Appendix*, Fig. S4*A*). We tentatively assigned enhancers to individual nearest genes. To reasonably limit arbitrariness in gene assignment, we only considered genes with a mapped TSS within 15 kb of an enhancer. The genes nearest to stimuli-inducible enhancers showed a greater transcriptional response than genes annotated to background enhancers (Fig. 3*A* and *SI Appendix*, Fig. S4*A*). This indicates that differential enhancer activity is directly reflected in gene expression levels. Importantly, genes associated with inducible enhancers belonged to several pathways, including cell signaling and blood cell migration such as *SGK1, CALCRL, CXCL2, CXCL5,* and *ITGA4* (Fig. 2*C* and *SI Appendix*, Table S4). We found a clear correlation between the number of genes showing differential expression and the changes in enhancer activity. Sixteen percent of genes nearest to de novo enhancers display ≥1.5-fold induction after dmPGE_2_ treatment, compared to 9% of enhanced and 3% of background enhancers (Fig. 3*B* and *SI Appendix*, Fig. S4*B*). In total, 83 (16%) of the 535 up-regulated genes were regulated by at least one stimuli-responsive enhancer (Fig. 3*C* and *SI Appendix*, Fig. S4*C*). This demonstrated that dmPGE_2_-mediated activation of stimuli-responsive enhancers correlates with modulation of gene expression.

To gain a better understanding of the role of pCREB at inducible enhancers, we further segmented enhancers based on the presence or absence of pCREB after dmPGE_2_ treatment. We found that 208/498 (42%) de novo, 210/456 (46%) enhanced, and 125/486 (26%) background enhancers to contain at least 1 pCREB ChIP-Seq peak after dmPGE_2_ treatment. Chromatin accessibility and MTF binding increased more profoundly, but not exclusively, at pCREB^+^ stimuli-inducible enhancers (Fig. 3*D*). The results support a prominent, but not restricted, role for pCREB in regulating gene expression through binding at inducible enhancers. Additionally, pCREB^+^ stimuli-responsive enhancer showed greater transcriptional changes than those without pCREB (Fig. 3*E* and *SI Appendix*, Fig. S4*D*). Together, our data suggested that increased enhancer chromatin accessibility and the cooperative binding of MTFs and STFs at stimuli-responsive regulatory regions drive transcriptional induction.

### Inducible Enhancers Retain Accessible Nucleosomes after Inflammatory Stimulation.

Various studies showed that TFs preferentially bind to sites of open, accessible chromatin (31). However, recent work indicated that accessible chromatin may not necessarily represent nucleosome-depleted regions (32, 33). To test whether greater chromatin accessibility at stimuli-responsive enhancers is due to nucleosome depletion, we performed micrococcal nuclease sequencing (MNase-Seq). This allowed us to map nucleosome positions and nucleosome occupancy changes after dmPGE_2_ stimulation. Because nucleosomes have varying sensitivities to enzymatic digestion, MNase titrations were performed to obtain a comprehensive map of the nucleosome landscape within a genome (32–34). While comparing occupancy profiles between individual titration points provides insights on nucleosomal accessibility, the combinatorial analysis of all titration points within a given condition generates a complete view of the nucleosome organization (32) (*SI Appendix*, Fig. S5*A*). We prepared native nuclei from HSPCs treated with either DMSO or dmPGE_2_ and exposed them to increasing units of MNase. We selected four digestion points that generated increasingly larger fractions of mononucleosomal-size DNA. Mononucleosomal fractions comprised around 10%, 25%, 50%, and 75% of the input chromatin, respectively (*SI Appendix*, Fig. S5*B*). Individual MNase titration profiles within a condition, as well as pooled average nucleosome occupancy profiles, revealed a TSS pattern similar to those previously reported (32) (*SI Appendix*, Fig. S5 *C* and *D*). We observed lower average nucleosome occupancy at TSS-proximal regions of dmPGE_2_-responsive genes after treatment (*SI Appendix*, Fig. S5*E*). This inverse correlation aligns with former studies which identified high levels of transcription to be concomitant with nucleosome eviction at the TSS, as elongation by RNA Polymerase II is thought to disrupt the nucleosomal organization (35, 36).

**Fig. 4.**
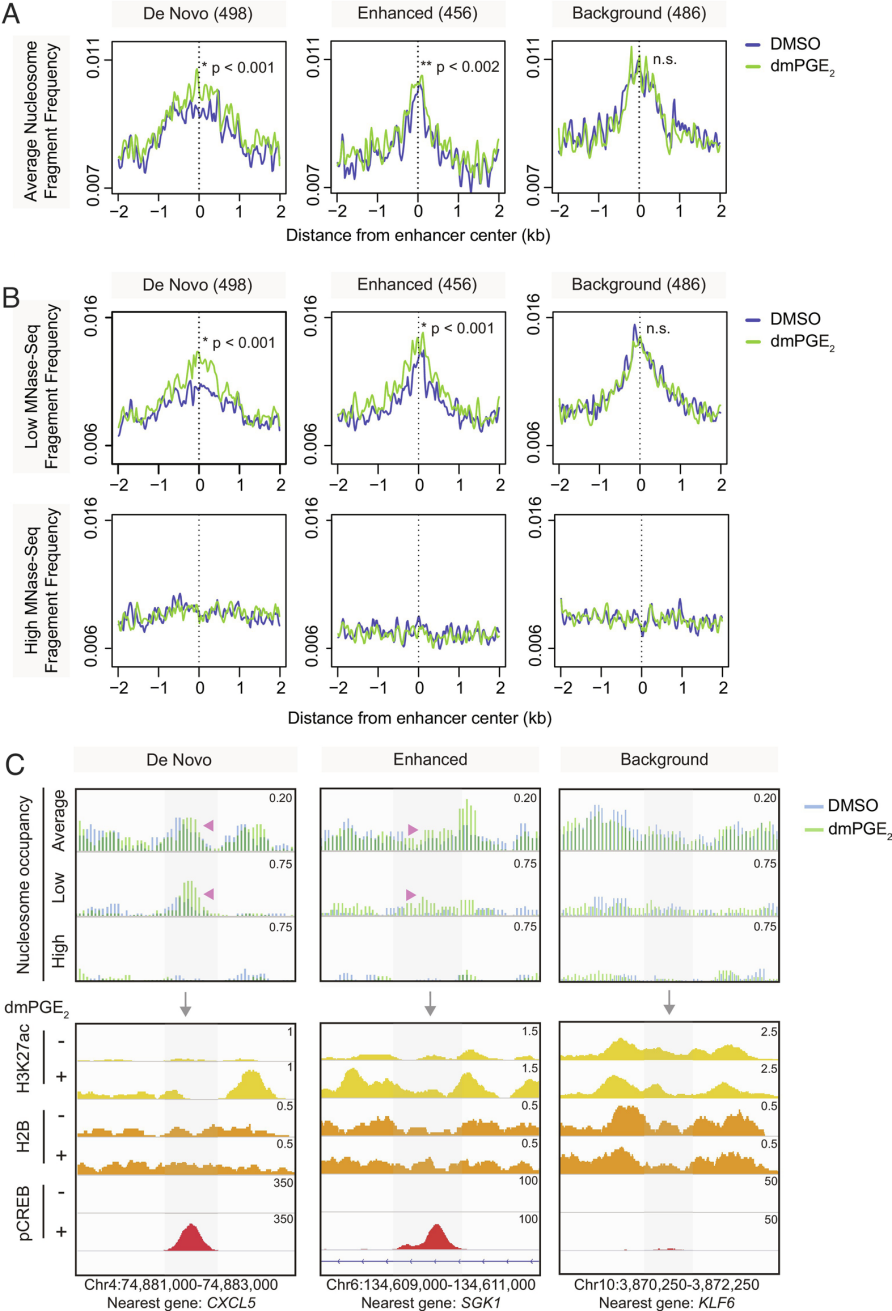
DmPGE_2_-responsive enhancers retain accessible nucleosomes after stimulation. (*A*) Average nucleosome occupancy profiles at stimuli-responsive and background enhancers from 4 MNase titration points (n = 3 biologically independent MNase-Seq experiments). (*B*) Nucleosome profiles of low and high MNase-Seq at stimuli-responsive and background enhancers (n = 3 biologically independent experiments). *P*-values by Wilcoxon rank-sum test. (*C*) Nucleosome fragment frequency and enrichment of H3K27ac, H2B, and pCREB at three representative stimuli-responsive and background enhancers. Genomic location of presented window and nearest gene is indicated at the bottom of the panel. Genomic location of presented window and enhancer nearest gene is indicated at the bottom of the panel. For all analyses presented here, a randomly sampled set of background enhancers (486) was used.

When assessing the nucleosome organization at inducible enhancers, we found that these regions presented in high nucleosome occupancy states when compared to active promoters (Fig. 4*A*). Although high occupancy regions are traditionally thought of as “closed”, recent work indicated that accessible regulatory regions can retain nucleosomes (32–34). We determined whether nucleosomes were repositioned or evicted at dmPGE_2_-responsive enhancers following stimulation. No decrease in average nucleosome occupancy was observed at stimuli-responsive enhancers. Inducible enhancers demonstrated higher nucleosome occupancy after dmPGE_2_ treatment (Fig. 4*A*). This was not observed at background enhancers, indicating specificity of the phenomena to dmPGE_2_-inducible enhancers. Our data suggest that stimuli-responsive enhancers retained nucleosomes upon activation, rather than remodeled to a nucleosome-free organization via nucleosome eviction.

Nucleosome profiles from individual MNase titration points can be leveraged to determine how MNase sensitivity changes in specific regions (*SI Appendix*, Fig. S5*A*). Light MNase conditions preferentially release accessible and unstable nucleosomes, whereas stable nucleosomes are more resistant to enzyme digestion and only released at higher MNase concentrations (32–34). We found greater low MNase-Seq signal at inducible enhancers after dmPGE_2_ stimulation (Fig. 4 *B*, *upper panel*), whereas high MNase digestion degraded the nucleosomal DNA fragments (Fig. 4 *B*, *bottom panel*). No changes in low MNase sensitivity were observed in background enhancers. Greater low MNase sensitivity after dmPGE_2_ treatment indicates a higher presence of MNase accessible nucleosomes. This shows that nucleosomes retained within inducible enhancers gained low MNase sensitivity upon stimulation.

To ensure that the low MNase-Seq signal represents nucleosomes, we performed ChIP-Seq for the core histones H2B and H4 (Fig. 4*C* and *SI Appendix*, Fig. S6*A*). Inducible enhancers were not depleted of core histones prior to or after dmPGE_2_ stimulation. This is in line with our MNase-Seq data and suggested that nucleosomes are indeed present, and retained at, dmPGE_2_-responsive enhancers. In contrast to our expectations that increased chromatin accessibility at inducible enhancers (Fig. 2*B*) resulted from nucleosome displacement or eviction, our MNase-Seq and ChIP-Seq data revealed that stimuli-responsive enhancers retained accessible nucleosomes upon activation.

**Fig. 5.**
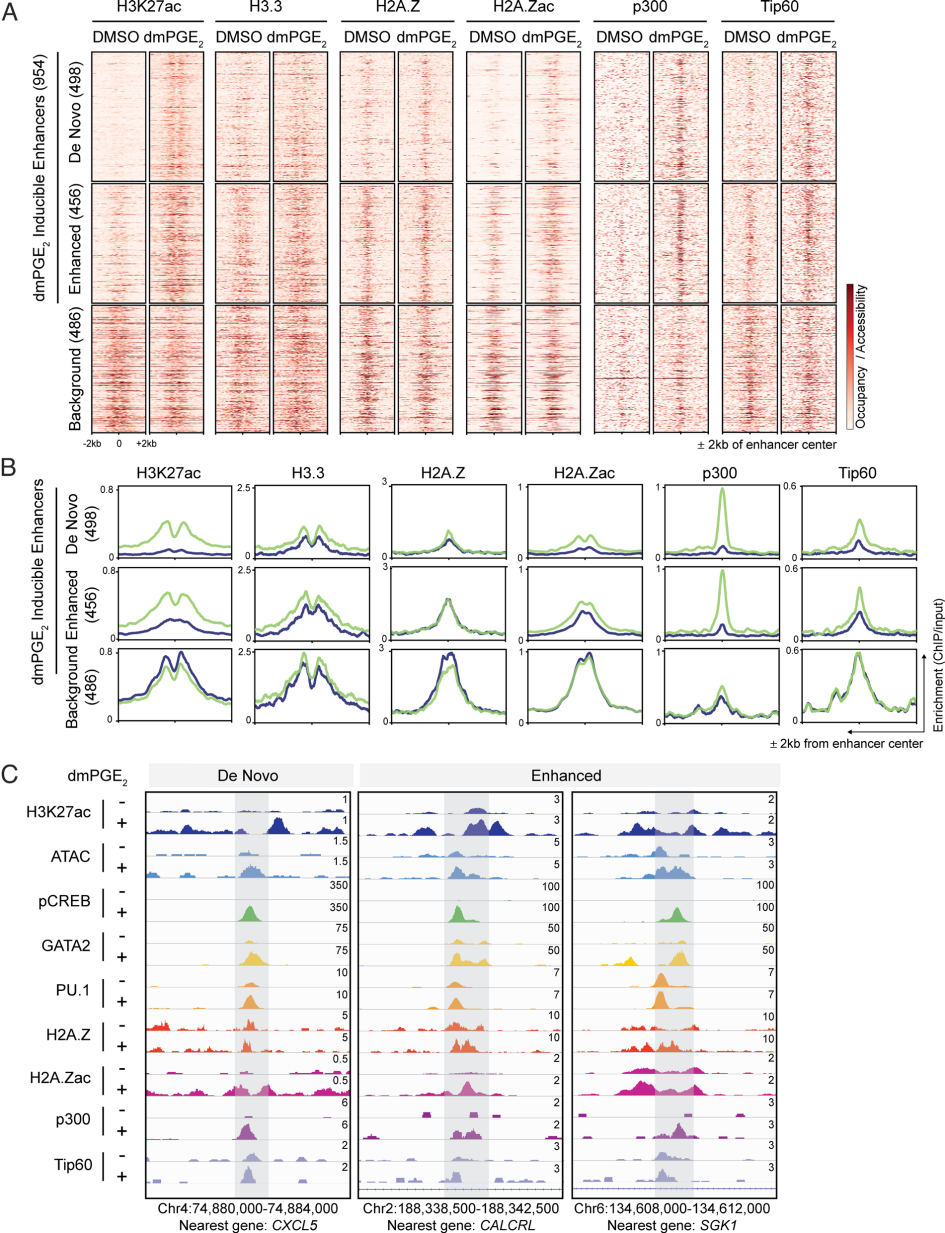
Modification of H2A.Z-variant-accessible nucleosomes at stimuli-responsive enhancers by HATs p300 and Tip60. (*A*) Heat maps of H3.3, H2A.Z, H2A.Zac, p300, and Tip60 binding at enhancers before and after dmPGE_2_ treatment. H3K27ac-enriched regions identified by ChIP-Seq are classified as de novo, enhanced, or background enhancers according to the change in H3K27ac levels observed following dmPGE_2_ stimulation (n = 2 biologically independent experiments). (*B*) Average enrichment profiles of histone variants and HATs before and after dmPGE_2_ treatment in de novo, enhanced, or background enhancers. (*C*) Enrichment of chromatin binding factors and TFs in response to dmPGE_2_ at three representative stimuli-response enhancers. Genomic location of presented window and enhancer nearest gene is indicated at the *Bottom* of the panel. For all analyses presented here, a randomly sampled set of background enhancers (486) was used.

Based on the accessible nucleosomes at enhancers, we next evaluated the relationships between nucleosomes and binding of pCREB at inducible enhancers. A total of 10,169/23,386 (43%) of pCREB binding sites uniquely present after dmPGE_2_ treatment are located within the 25,998 active enhancers identified in HSPCs. We observed enrichment of phased nucleosomes at the summit of dmPGE_2_-unique pCREB peaks located within enhancers, both before and after dmPGE_2_ treatment (*SI Appendix*, Fig. S7*A*). To exclude the possibility that the MNase-Seq fragments observed at pCREB sites within enhancers represent nonhistone proteins protecting from MNase digestion, we assessed fragment size distribution (*SI Appendix*, Fig. S6*B*). We found a fragment periodicity that is characteristic for MNase-digested nucleosomes (37, 38). The majority of the MNase-Seq fragments that coincide with pCREB binding was 148 base pairs (bp) in length. This corresponds to precisely trimmed nucleosome core particles. Subnucleosomal peaks showed a clear ~10 bp periodicity that reflects the accessibility of DNA as it is wound along the surface of the histone octamer (39). This analysis suggested that MNase-Seq fragments mapping to pCREB sites within enhancers represent nucleosomes. Looking specifically at pCREB^+^ stimuli-responsive enhancers, we observed that the effects of dmPGE_2_ on nucleosome occupancy and low MNase sensitivity described earlier are further amplified at pCREB^+^ regions (*SI Appendix*, Fig. S7 *B* and *C*). Assessment of individual loci confirmed pCREB binding at stimuli-responsive enhancers that show greater low MNase signal and increased nucleosome occupancies after dmPGE_2_ treatment (Fig. 4*C*). Together, the data demonstrated that accessible nucleosomes are retained at dmPGE_2_-responsive enhancers and that these nucleosomes are not prohibitive of pCREB binding.

### Modification of H2A.Z-Variant-Accessible Nucleosomes at Stimuli-Responsive Enhancers.

**Fig. 6.**
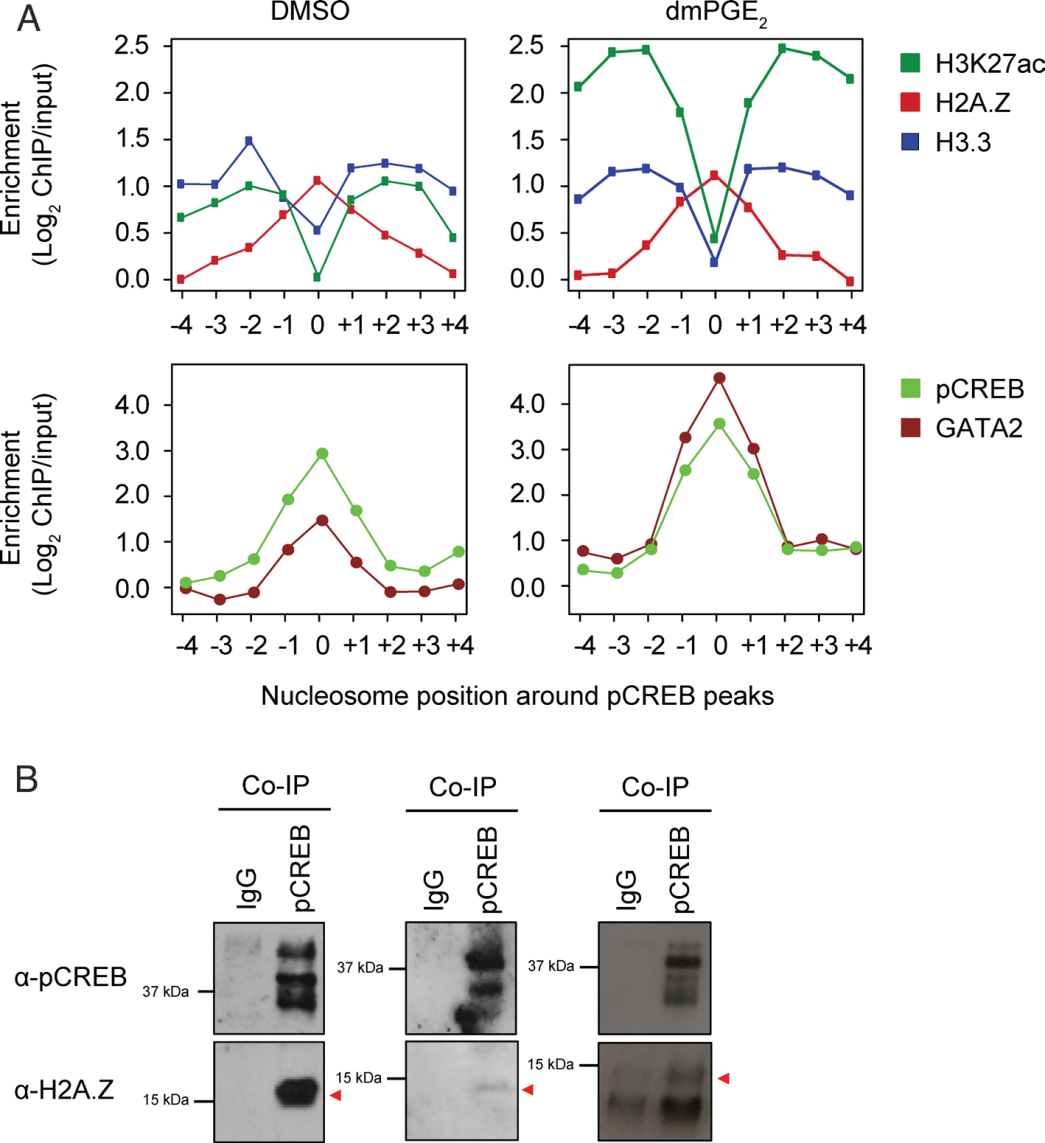
H2A.Z-variant nucleosomes cooccupy pCREB binding sites at stimuli-responsive enhancers. (*A*) Enrichment of histone variants at nucleosome positions surrounding pCREB peaks within enhancers before and after dmPGE_2_ stimulation. Position 0 indicates the nucleosome overlapping with pCREB peak centers. (*B*) Co-IP showing that pCREB associated with H2A.Z in U937 myeloid leukemia cells (n = 3 biologically independent experiments).

We hypothesized that nucleosome at enhancers may exert important roles in pCREB binding. Nucleosome-driven TF binding has been observed for several other stress-responsive TFs (40). To understand the conformational changes that underlie increased low MNase sensitivity of enhancer nucleosomes after dmPGE_2_ stimulation, we investigated other mechanisms that influence accessibility to nucleosomal DNA. Weakening of internucleosomal interactions by covalent modifications of histones as well as the introduction of histone variants increases DNA accessibility (41). The histone variants H2A.Z and H3.3 can be incorporated in replication-independent manners and are associated with enhancers (42–44). Once incorporated, these noncanonical histones make for destabilized “fragile” nucleosomes (45). We assessed histone-variant abundance at regulatory regions in the presence and absence of dmPGE_2_ stimulation through ChIP-Seq analysis for H2A.Z and H3.3. We observed a positive correlation between histone-variant deposition and enhancer activity, with more profound presence of H2A.Z and H3.3 at higher H3K27ac levels (Fig. 5 *A* and *B*). The histone variants were also found to occupy slightly different sites within enhancers. H2A.Z localized more central to enhancers, the region where TFs bind, whereas H3.3-variant nucleosomes followed a more dispersed pattern and localized to the flanks of enhancers in a profile similar to H3K27ac (Fig. 5 *A* and *B* and *SI Appendix*, Fig. S6*C*). We noted minimal changes in H2A.Z enrichment following dmPGE_2_ stimulation. We did observe incorporation of the histone-variant H3.3 in the nucleosomes of enhancers (Fig. 5 *A* and *B*). When specifically assessing histone variants at pCREB^+^ stimuli-responsive enhancers, we found that pCREB binding directly overlaps with H2A.Z-variant nucleosomes (Fig. 6*A*). To confirm interaction of pCREB with H2A.Z-variant nucleosomes, we performed complex immunoprecipitation (Co-IP) for pCREB and found association between the TF and H2A.Z histones (Fig. 6*B*). These results indicated that pCREB overlaps with H2A.Z and suggest that DNA binding of the TF is not prohibited by H2A.Z-variant nucleosomes.

H2A.Z is associated with both gene repression and activation (46). This dual function is attributed to posttranslational regulation of the histone variant. Different H2A.Z histone tail modifications recruit distinct interactors that mediate varying transcriptional outputs. H2A.Z acetylation is associated with active transcription and dynamically regulated in response to environmental signals (47, 48). Acetylation of H2A.Z occurs at active regulatory regions where it promotes nucleosome destabilization and an open chromatin conformation. The histone acetyltransferases (HATs) p300 and Tip60 that acetylate H2A.Z are known interactors of pCREB (45, 49–53). Both chromatin remodelers hold important roles in hematopoietic stem cell fate (45, 51–53). Whereas Tip60 alone is not sufficient to acetylate H2A.Z, p300 can rapidly and effectively acetylate H2A.Z-containing nucleosomes on its own (50). Hence, we assessed whether acetylation of H2A.Z-variant nucleosomes at stimuli-responsive enhancers underlies increased MNase sensitivity and nucleosome accessibility after dmPGE_2_ treatment. We found specific H2A. Zac enrichment at stimuli-inducible enhancers, whereas this was not observed in background enhancers (Fig. 5 *A*–*C*). We furthermore noted that dmPGE_2_ increased the abundance of both p300 and Tip60 at enhancers (Fig. 5 *A*–*C*). pCREB interacts with a variety of chromatin remodelers, including p300 and Tip60 (50, 54). Binding patterns of two HATs at enhancers were highly similar to pCREB after dmPGE_2_ treatment (Fig. 5*B*) and are suggestive of complex interaction at enhancers. Together, our data revealed that pCREB binds to H2A.Z-variant nucleosomes within enhancers following dmPGE_2_ stimulation. pCREB binding at stimuli-responsive enhancers is accompanied by nucleosome remodeling through H2A.Z acetylation, likely mediated through interaction of CREB with p300 and Tip60. H2A.Z acetylation at enhancers may underlie increased enhancer accessibility, allowing additional chromatin factors to engage and ultimately drive acute gene expression changes.

There has been a significant body of literature that suggests the importance of p300 in mediating H2A.Z acetylation (13, 49, 50, 55). CBP is highly homologous to p300 and demonstrates over 60% overlap at the amino acid level (56). Several studies suggest both comparable and distinct contributions of p300 and CBP with regard to protein acetylation (57). Here, we do not discriminate against potential contribution of CBP and believe both p300 and CBP could acetylate H2A.Z to transcriptionally activate dmPGE_2_ target genes in HSPCs. Only recently have a small number of studies implicated a role for Tip60 in blood development (51, 58). As such, the exact role of Tip60 in regulating blood cells, specifically during transcriptional activation of stress-induced hematopoietic regeneration, remains elusive. To document the role of Tip60 in modulating the transcriptional effects of dmPGE_2_–pCREB signaling, we followed two separate loss-of-function approaches to knock down TIP60 expression and assess downstream transcriptional effects of dmPGE_2_ as compared to DMSO. We reduced *TIP60* expression by siRNA and with the Tip60 inhibitor NU9056 (51, 58). We observed a moderate yet statistically significant inhibition of dmPGE_2_ induction of target genes under siRNA-mediated knockdown of *TIP60* in CD34^+^ HSPCs (*SI Appendix*, Fig. S8 *A* and *B*). Additionally, NU9056 treatment of U937 cells demonstrated a substantial decrease of H2A.Z acetylation and under such conditions, dmPGE_2_ target genes appeared to show a reduction in gene induction compared to their respective control condition (*SI Appendix*, Fig. S8 *C* and *D*). While reduction in PGE2-mediated gene expression upon loss of Tip60 is moderate, we did not anticipate huge drop given very high fold-change observed under normal conditions. However, taken together, inhibition of Tip60 demonstrated a statistically significant trend toward compromised transcriptional responsiveness during dmPGE_2_ signaling.

## Discussion

PGE2 is an important regulator of HSPC homeostasis. The distinct molecular mechanisms through which PGE_2_ and its stable derivative dmPGE_2_ affect HSC function are critical to understand yet remain elusive. Here, we find that the TF CREB is a key player in the acute transcriptional response to dmPGE_2_ by binding to, and activating, distal regulatory elements. Specifically, we find that pCREB binds to H2A.Z-variant nucleosomes that are retained within stimuli-induced, active enhancers and is concomitant with acetylation of these histones. Upon dmPGE_2_ treatment, intensity of both H2A.Z and H2A.Zac increases specifically at pCREB-bound, dmPGE_2_-inducible enhancers, suggesting that acetylation of H2A.Z may likely increase on-chromatin interaction of pCREB and H2A.Z. Such interactions between pCREB and H2A.Z may further increase H2A.Z acetylation of enhancer nucleosomes to synergistically promote local chromatin accessibility, which may subsequently help recruit and/or stabilize other HSPC-specific TFs such as GATA2 to stimulate gene transcription and strengthen blood stem cell fate.

CREB is a ubiquitously expressed nuclear, basic leucine zipper (bZIP) TF that regulates over 5,000 genes in the mammalian genome. This includes genes controlling proliferation, differentiation, and cell survival (59). The TF is activated by a wide variety of environmental stimuli and is an important regulator of cellular responses to stress. Most studies focused on promoter-proximal effects of CREB binding. We showed that inducible gene expression is characterized by binding of pCREB at TSS-distal enhancer regions. Regulation of transcriptional responses through binding of CREB at enhancers has also been observed in pancreatic beta cells (60). Our data show that CREB employs distinct mechanisms to regulate steady-state versus inducible gene expression.

Genome-wide assessment of the epigenetic landscape revealed that dmPGE_2_ works within the predetermined enhancer repertoire of HSPCs. dmPGE_2_ stimulation activates a set of preexisting H3K4me1^+^ enhancers through chromatin reorganization. dmPGE_2_-responsive enhancers rapidly gain accessibility and TF binding. Our work complements the studies that showed that STFs localize to binding sites adjacent to master regulators (30, 61). Although we did not identify the surfacing of latent enhancers, i.e., genomic regulatory elements devoid of TFs and enhancer marks in unstimulated cells (27), a 2-h pulse of dmPGE_2_ may be too short to allow for partial reprogramming of the available cis-regulatory landscape. Latent enhancer activation may also be more associated with differentiated cells rather than stem and progenitor populations (27).

We found stimuli-driven enrichment of the HSPC-specific MTFs GATA2 and PU.1 at inducible enhancers, especially those that gained pCREB binding after 2 h of treatment. Cofactor driven binding is common among nonpioneer TFs. It was only recently implied that interaction between TFs can enhance pioneer factor binding at previously sampled target sites (62–64). Many studies have described the vital role of GATA2 in establishing the regulatory landscape for STFs in HSPCs (30, 65). GATA2 facilitates enhancer–promoter loop formation (66), yet few have proposed signaling factors to play a role in GATA2 binding and recruitment to chromatin. We suggest that GATA2 occupancy may be directed and stabilized through cooperativity with STFs at stimuli-responsive enhancers. Our work supports a model where pioneer factor occupancy at specific subsets of enhancers is partially determined by engagement with signaling specific cofactors.

An open chromatin structure surrounding TF binding sites constitutes a prerequisite for transcriptional regulation. Our results show that inducible enhancers rapidly gain accessibility and affinity for STFs and MTFs. While open chromatin regions are presumed nucleosome-depleted regions, we find that dmPGE_2_-responsive enhancers exhibit a high nucleosome occupancy both prior to and after stimulation of HSPCs. Moreover, retained enhancer nucleosomes are not prohibitive of STF binding. pCREB occupancy at enhancers overlaps with well-positioned nucleosomes, suggesting that pCREB can initiate chromatin engagement and access binding sites organized within a positioned nucleosome. Our work indicates that accessible nucleosomes at enhancers may facilitate cooperativity between STFs and MTFs to ensure rapid transcriptional induction. Retention of MNase-accessible nucleosomes at regulatory elements was proposed to play a crucial role in hormone signaling and tissue-specific gene activation (33, 40). Retained nucleosomes likely stabilize the interaction of TFs with DNA by facilitating interactions between TF-associated factors, such as chromatin remodeling complexes and histone tails (40).

Except for pioneer factors, most TFs are thought to be unable to bind nucleosomal DNA. Although CREB is not traditionally described as a pioneer factor, novel studies revealed the ability of CREB to open chromatin (67). CREB was identified as a TF that displays an orientated, asymmetric, binding preference near the dyad axis of the nucleosome when engaging nucleosomal DNA (64, 65). We hypothesize that MNase-accessible nucleosomes within stimuli-responsive enhancers enable cooperative TF binding for rapid gene activation.

Nucleosomes retained within stimuli-inducible enhancers were epigenetically premarked by the histone-variant H2A.Z. Although the precise function of H2A.Z at enhancers at remains unclear, H2A.Z is an important regulator of enhancer activity in response to stimuli. H2A.Z-rich enhancers display higher chromatin accessibility and gene induction by promoting RNA polymerase II recruitment (42, 46, 68). In contrast to hormone stimulation, which was found to increase H2A.Z incorporation at enhancer nucleosomes (69), we found limited changes in H2A.Z distribution after dmPGE_2_ treatment. Instead, we noted that H2A.Z-variant nucleosomes undergo histone tail acetylation following dmPGE_2_ stimulation. Acetylated forms of H2A.Z are associated with an open chromatin conformation and directly regulate transcription of enhancer RNAs (50, 54, 55, 58, 70, 71). Our work implies that dmPGE_2_-inducible H2A.Z acetylation underlies increased low MNase sensitivity and enhanced nucleosome accessibility at stimuli-responsive enhancers following dmPGE_2_ treatment. We found that changes in posttranslational acetylation of H2A.Z at stimuli-responsive enhancers correlate with gene expression changes, indicating that H2A.Zac is a prerequisite for appropriate transcriptional induction following dmPGE_2_ stimulation.

Labile, H2A.Z-marked nucleosomes do not present an obstacle for pCREB binding but may facilitate TF binding and enhancer activity. We suggest that a critical feature of pCREB is the recruitment of remodelers that opens the local nucleosome structure through H2A.Z acetylation in enhancers. pCREB interacts with a variety of chromatin remodelers. This includes the HATs p300 and Tip60, both known to interact with H2A.Z and catalyze its acetylation (50, 54, 58). The recruitment of p300 to chromatin in a stimulus-dependent manner observed here is consistent with interactions between chromatin remodelers and other STFs (72, 73). Localization of p300 is concomitant to pCREB binding, suggesting pCREB–p300 complex formation at enhancers upon dmPGE_2_ stimulation. pCREB-facilitated recruitment of Tip60 to enhancers complements previously described observations of Tip60 binding to a subset of enhancers and acetylating H2A.Z to promote expression of HSC genes (58, 74). The acetylation of H2A.Z-variant enhancer nucleosomes may create a chromatin environment permissive of enhancer activity and transcription.

This study reveals how specific genomic reorganization at a stimuli-responsive group of enhancers is directly translated into regulatory element activation and transcriptional induction. Our findings support a model where STFs and MTFs cooperate with nucleosomes to regulate the activity of cis-regulatory elements that mediate adequate responses to environment signals. While the combination of cooperative lineage-specific MTF and inducible STF binding provides context and responsiveness to external stimuli, histone-variant nucleosomes retained within inducible enhancers may facilitate TF binding by stabilizing chromatin complexes. Subsequent acetylation of histone-variant nucleosomes by TF-associated nucleosome remodelers creates the accessible nucleosome landscape required at active transcriptional enhancers to ensure strong gene activation.

Our research previously identified PGE_2_ as a potent regulator of HSPC fate, which became the first compound to move from a zebrafish screen to the clinic (6). Starting with the discovery of PGE_2_ in 2007, we have since reported its scientific journey from the bench to the bedside, describing the potential of PGE_2_ to improve the success of blood stem cell transplantation in clinical trials (5, 6, 8). However, the exact mechanisms through which PGE_2_ regulates HCPC fate have remained elusive. This study demonstrates the mechanism by which inflammatory lipids such as PGE_2_ alter chromatin and HSPC engraftment to improve transplantation. PGE_2_ and its stable derivate dmPGE_2_ mediate chromatin accessibility at stimuli-inducible enhancers through histone-variant H2A.Z acetylation to promote master TF binding. The acute changes at enhancer elements drive reinforcement in the expression of genes involved in stem cell fate and engraftment. Higher expression of such genes may drive selection of stem cells with greater potency and long-term activity in the natural bone marrow niche after transplantation. As PGE_2_ is essential for various types of stem cells (75), this mechanism may improve stem cell function in organ systems beyond those of the blood. We propose that other stress-responsive signals besides PGE_2_, such as estrogen signaling, may utilize similar mechanisms through acetylation of histone variants (48, 68). Plasticity of chromatin architecture achieved through modification of labile nucleosomes provides a rapid mechanism to promote stem cell fate through a variety of stress-activated signaling cues over a large extent of tissue types.

## Materials and Methods

### Expansion of CD34^+^ Cells.

Human CD34^+ ^(HSPCs), isolated from granulocyte-colony-stimulating factor (G-CSF) mobilized peripheral blood of healthy volunteers, were purchased from the Fred Hutchinson Cancer Research Center. The cells were maintained in suspension culture as previously described by Trompouki et al. (30).

### Cell Culture.

U937 cells were maintained in suspension culture in RPMI-1640 supplemented with 10% (v/v) heat-inactivated fetal bovine serum, 1× GlutaMax, and 1% penicillin–streptomycin at 37° in a humidified atmosphere of 5% CO_2_.

### dmPGE_2_ Treatment.

16,16-dimethyl PGE2 was purchased, reconstituted in DMSO from Cayman Chemicals (cat. #14750), aliquoted, and stored in −80 °C until use. Cells were counted, collected, and resuspended in StemSpan medium with 2% PenStrep (CD34+ Cells) or RPMI with 1% penicillin–streptomycin, but in the absence of additional cytokines or growth factors. The cells were treated with 10 μM dmPGE_2_ (Cayman chemicals) or DMSO (vehicle control) for 2 h.

### qPCR Analysis.

RNA was extracted using the RNeasy plus mini kit (Qiagen). cDNA synthesis was performed using the Superscript VILO (Invitrogen) and using equal amounts of starting RNA. The cDNA was analyzed with the Light Cycler 480 II SYBR green master mix (Applied Biosystems), and the QuantStudio 12K Flex (Applied Biosystems). All samples were prepared in triplicate. The PCR cycle conditions used are: (a) 95 °C for 5 min, (b) 95 °C for 10 s, 54 °C for 10 s, 72 °C for 15 s × 40 cycles. The analysis of Ct values was performed using 2^-ΔΔT method. Primers used are listed in *SI Appendix*, Supplemental Materials and Methods.

### Western Blotting and (Co-IP).

These experiments are described in detail in *SI Appendix*, Supplemental Materials and Methods.

### Next-Generation Sequencing.

Detailed description of RNA-Seq, ChIP-Seq, ATAC-Seq, and MNase-Seq methods is available in *SI Appendix*, Supplemental Materials and Methods.

## Supplementary Material

Appendix 01 (PDF)

## Data Availability

Next Gen Sequencing Data data have been deposited in GEO DataSets (76).
