# Supplementary material for: PGE2 alters chromatin through H2A.Z-variant enhancer nucleosome modification to promote hematopoietic stem cell fate
Source: Proc Natl Acad Sci U S A. 2023 May 1;120(19):e2220613120. doi: 10.1073/pnas.2220613120 (PMC10175842; doi:10.1073/pnas.2220613120)
Supplement: Supplementary file 1 — Appendix 01 (PDF) [file pnas.2220613120.sapp.pdf]

Supplemental figures with legends

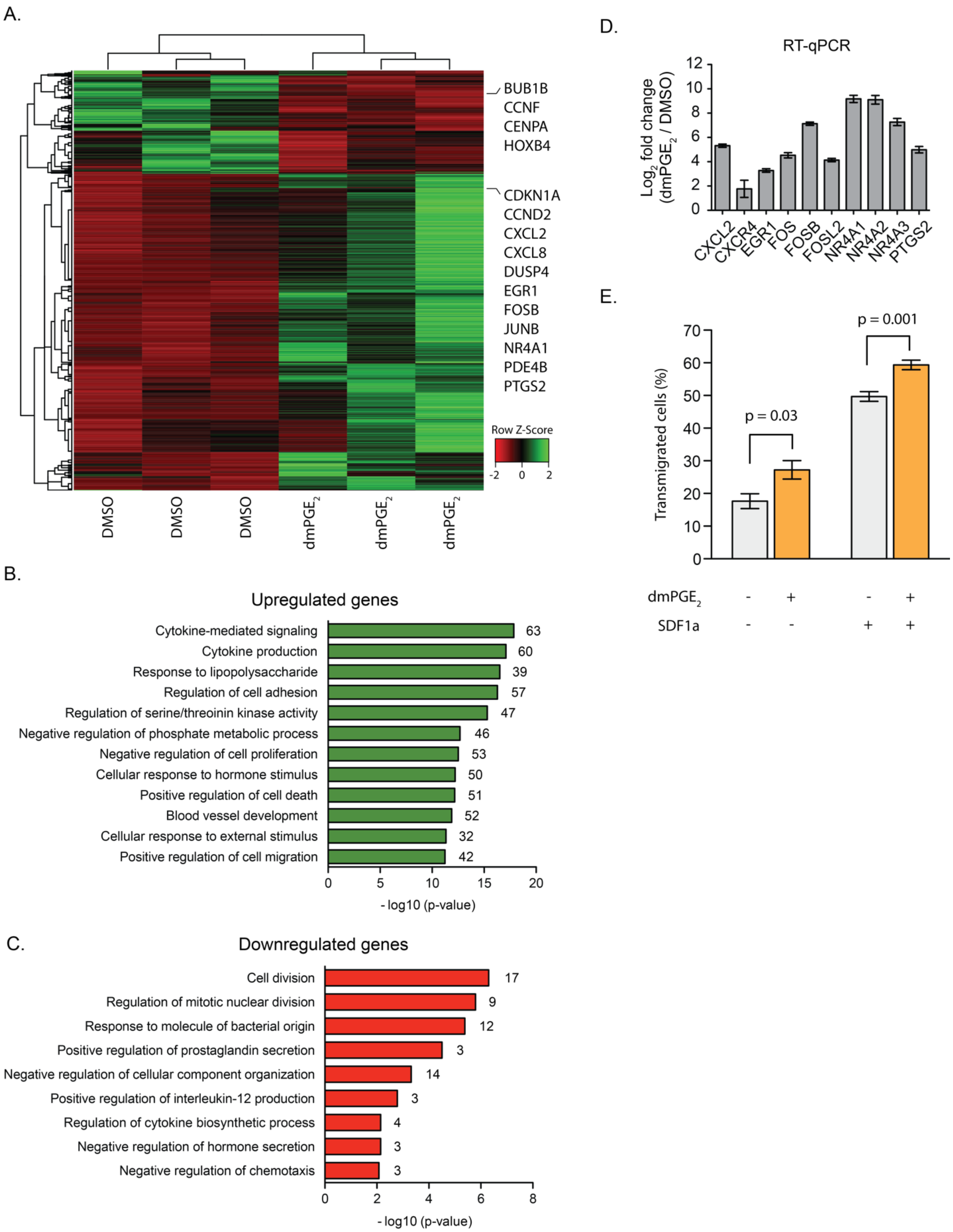
A.
BUB1B
CCNF
CENPA
HOXB4
CDKN1A
CCND2
CXCL2
CXCL8
DUSP4
EGR1
FOSB
JUNB
NR4A1
PDE4B
PTGS2
Row Z-Score
-2 0 2
DMSO
DMSO
DMSO
dmPGE2
dmPGE2
dmPGE2
B.
Upregulated genes
Cytokine-mediated signaling 63
Cytokine production 60
Response to lipopolysaccharide 39
Regulation of cell adhesion 57
Regulation of serine/threoinin kinase activity 47
Negative regulation of phosphate metabolic process 46
Negative regulation of cell proliferation 53
Cellular response to hormone stimulus 50
Positive regulation of cell death 51
Blood vessel development 52
Cellular response to external stimulus 32
Positive regulation of cell migration 42
0 5 10 15 20
- log10 (p-value)
C.
Downregulated genes
Cell division 17
Regulation of mitotic nuclear division 9
Response to molecule of bacterial origin 12
Positive regulation of prostaglandin secretion 3
Negative regulation of cellular component organization 14
Positive regulation of interleukin-12 production 3
Regulation of cytokine biosynthetic process 4
Negative regulation of hormone secretion 3
Negative regulation of chemotaxis 3
0 2 4 6 8
- log10 (p-value)
D.
RT-qPCR
Log2 fold change (dmPGE2 / DMSO)
0 2 4 6 8 10 12
CXCL2 CXCR4 EGR1 FOS FOSB FOSL2 NR4A1 NR4A2 NR4A3 PTGS2
E.
Transmigrated cells (%)
0 10 20 30 40 50 60 70
p = 0.03
p = 0.001
dmPGE2 - + - +
SDF1a - - + +

Supplemental Figure 1. dmPGE$_2$ induces acute transcriptional responses in HSPCs. (A) Hierarchical clustering heatmap of FPKM values from differentially expressed genes (687) 2 hours post-treatment. DEG criteria: FPKM ≥ 1 after treatment; fold change ≥ 1.5 or ≤ 0.67 (n = 3 biologically independent experiments). (B, C) Gene Ontology (GO) term enrichment analysis of genes upregulated (C, in green) and down-regulated (D, in red) in CD34$^+$ HSPCs 2 hours post dmPGE$_2$ treatment. The number of genes associated with each GO term are shown at the end of the bar within the graph. P-values were calculates using hypergeometric test and Benjamini-Hochberg correction. (D) RT-qPCR in CD34$^+$ HSPCs of genes identified as differentially expressed by RNA-Seq (n = 3 biologically independent experiments; mean values ± SEM). (E) CD34$^+$ HSPCs were exposed to dmPGE$_2$ or DMSO for 2h cells after which the stimuli were washed out. Cells were then placed in the top chamber of the transwell system, with or without recombinant human SDF-1α in the bottom chamber. After 24 hours, cells migration to the bottom chamber was quantified as percentage of total cells seeded. (n = 3 biologically independent experiments; mean values ± SEM).

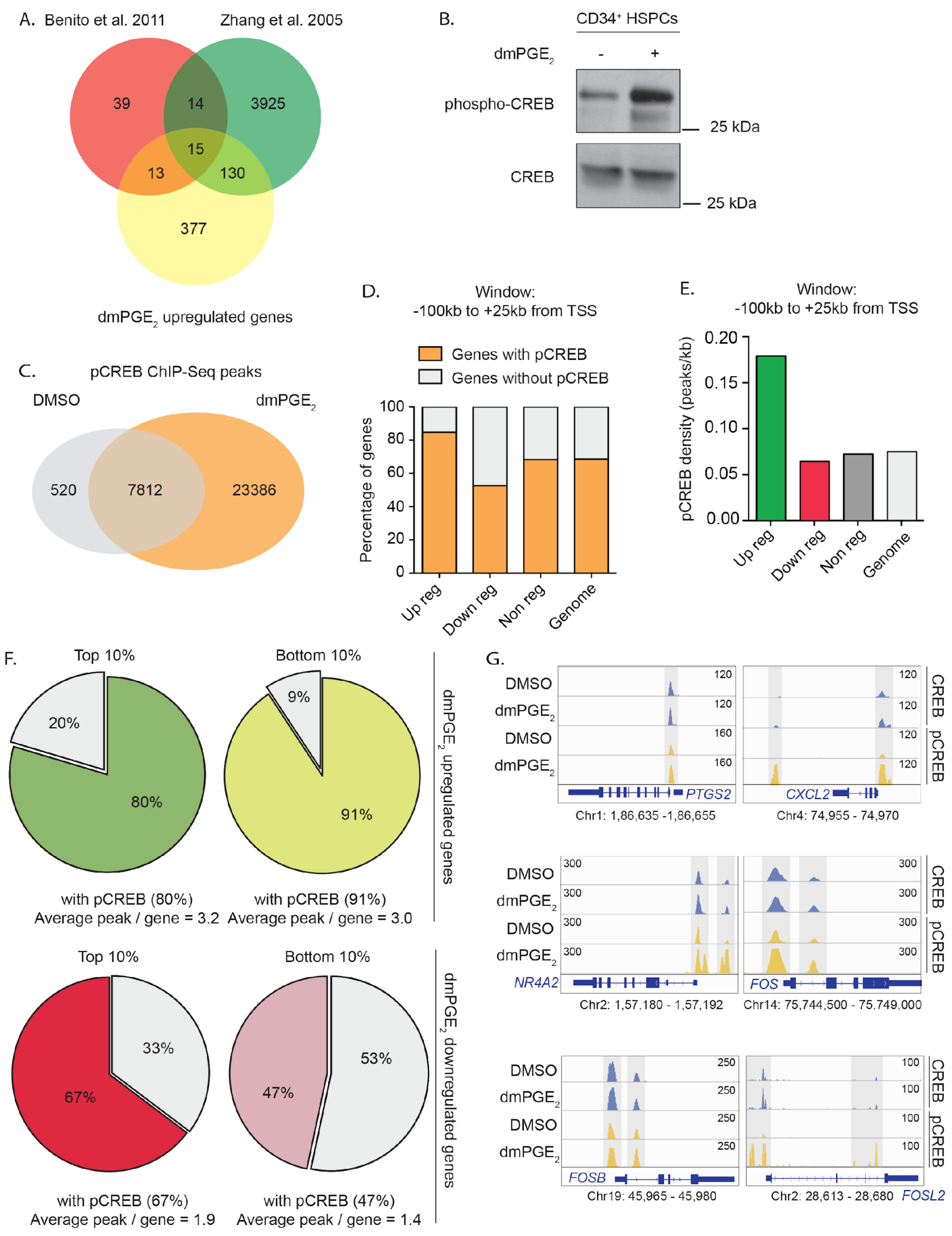
A. Benito et al. 2011
Zhang et al. 2005
39
14
3925
15
13
130
377
dmPGE2 upregulated genes
B.
CD34+ HSPCs
dmPGE2
-
+
phospho-CREB
25 kDa
CREB
25 kDa
C.
pCREB ChIP-Seq peaks
DMSO
dmPGE2
520
7812
23386
D.
Window:
-100kb to +25kb from TSS
Genes with pCREB
Genes without pCREB
Percentage of genes
Up reg
Down reg
Non reg
Genome
E.
Window:
-100kb to +25kb from TSS
pCREB density (peaks/kb)
Up reg
Down reg
Non reg
Genome
F.
Top 10%
20%
80%
with pCREB (80%)
Average peak / gene = 3.2
Bottom 10%
9%
91%
with pCREB (91%)
Average peak / gene = 3.0
dmPGE2 upregulated genes
Top 10%
33%
67%
with pCREB (67%)
Average peak / gene = 1.9
Bottom 10%
53%
47%
with pCREB (47%)
Average peak / gene = 1.4
dmPGE2 downregulated genes
G.
DMSO
dmPGE2
DMSO
dmPGE2
CREB
pCREB
PTGS2
Chr1: 1,86,635 -1,86,655
CXCL2
Chr4: 74,955 - 74,970
NR4A2
Chr2: 1,57,180 - 1,57,192
FOS
Chr14: 75,744,500 - 75,749,000
FOSB
Chr19: 45,965 - 45,980
Chr2: 28,613 - 28,680
FOSL2

Supplemental Figure 2. pCREB binds near differentially expressed genes. (A) Venn diagram showing overlap between upregulated genes (535) and previously identified CREB target genes. (B) Western blot analysis for phospho-CREB in $CD34^+$ HSPCs stimulated with vehicle control (DMSO) or $dmPGE_2$ for 2 hours. Total CREB protein was used as loading control. (C) Venn diagram showing overlap between pCREB peaks present in DMSO after $dmPGE_2$ stimulation, as identified by ChIP-Seq. (D) Number of genes containing at least one pCREB peak in the proximity after $dmPGE_2$ stimulation. pCREB peaks were assigned to a gene when located within a window from -100kb upstream of the transcription start site (TSS) to +25kb downstream of the TTS was considered (n = 2 biologically independent ChIP-Seq experiments). (E) Correlation between pCREB binding and gene expression in response to $dmPGE_2$. pCREB density was calculated by dividing the total number of pCREB peaks associated to each gene category (up-, down-, and nonregulated genes) by the total amount of base pairs that this category occupies in the genome. pCREB peaks were assigned to a gene when located from +100kb upstream of the TSS to +25kb downstream of the TTS. Peak density in the genome was calculated by considering random distribution of pCREB sites in the whole genome. (F) pCREB in $dmPGE_2$-response genes. Top and bottom 10% correspond to the 10% most upregulated and downregulated genes, respectively. pCREB peaks were assigned to a gene when located within a window from -5kb upstream of the transcription start site (TSS) to +5kb downstream of the TTS was considered (n = 2 biologically independent ChIP-Seq experiments). (G) Enrichment of CREB and pCREB binding at 6 representative $dmPGE_2$ response genes: *CXCL2*, *PTGS2*, *NR4A2*, *FOS*, *FOSL2* and *FOSB*. Gray bars indicate the CREB or pCREB peaks. Genomic location of presented window is indicated at the bottom of the panels.

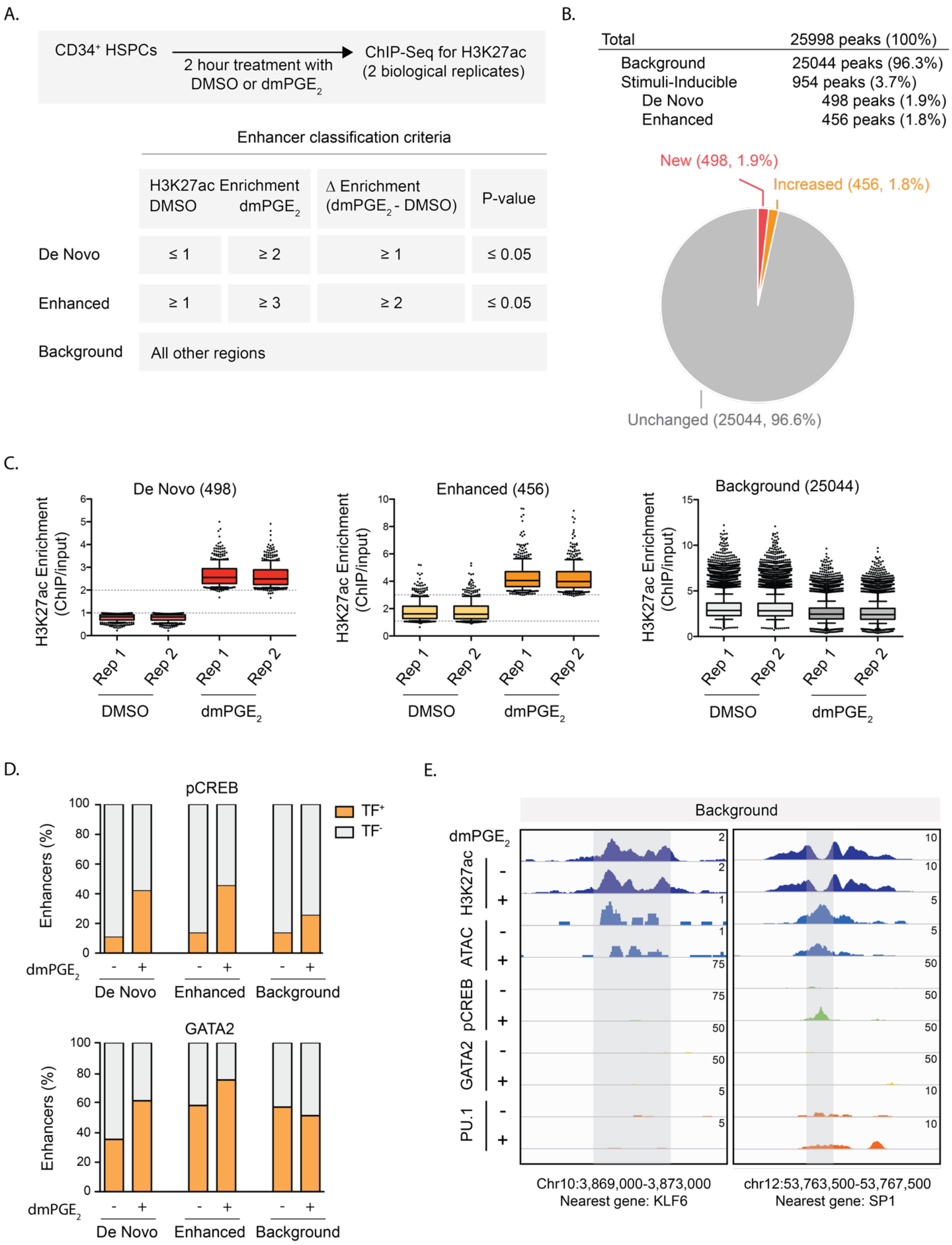
A.
CD34+ HSPCs
2 hour treatment with DMSO or dmPGE2
ChIP-Seq for H3K27ac (2 biological replicates)
Enhancer classification criteria
H3K27ac Enrichment
DMSO
dmPGE2
Δ Enrichment (dmPGE2 - DMSO)
P-value
De Novo ≤ 1 ≥ 2 ≥ 1 ≤ 0.05
Enhanced ≥ 1 ≥ 3 ≥ 2 ≤ 0.05
Background All other regions
B.
Total 25998 peaks (100%)
Background 25044 peaks (96.3%)
Stimuli-Inducible 954 peaks (3.7%)
De Novo 498 peaks (1.9%)
Enhanced 456 peaks (1.8%)
New (498, 1.9%)
Increased (456, 1.8%)
Unchanged (25044, 96.6%)
C.
De Novo (498)
Enhanced (456)
Background (25044)
H3K27ac Enrichment (ChIP/input)
Rep 1
Rep 2
DMSO
dmPGE2
D.
pCREB
GATA2
Enhancers (%)
TF+
TF-
dmPGE2
De Novo
Enhanced
Background
E.
Background
dmPGE2
H3K27ac
ATAC
pCREB
GATA2
PU.1
Chr10:3,869,000-3,873,000
Nearest gene: KLF6
chr12:53,763,500-53,767,500
Nearest gene: SP1

Supplemental Figure 3. Identification of stimuli-inducible enhancers in HSPCs. (A) Experimental set up and enhancer classification criteria. (B) Number of identified enhancer regions and their distribution into the different categories based on two independent replicate experiments. (C) H3K27ac enrichment levels at enhancers within each indicated category for 2 biologically independent replicate (rep) ChIP-Seq experiments. Dotted lines indicate cutoff values used for enhancer classifications. (D) Number of enhancers as percentage of total within each category containing enrichment for pCREB (upper panel) and GATA2 (lower panel). (E) Enrichment of histone mark, ATAC accessibility and transcription factor binding in response to $dmPGE_2$ at representative 2 background enhancers. Genomic location of presented window and nearest gene are indicated at the bottom of the panel.

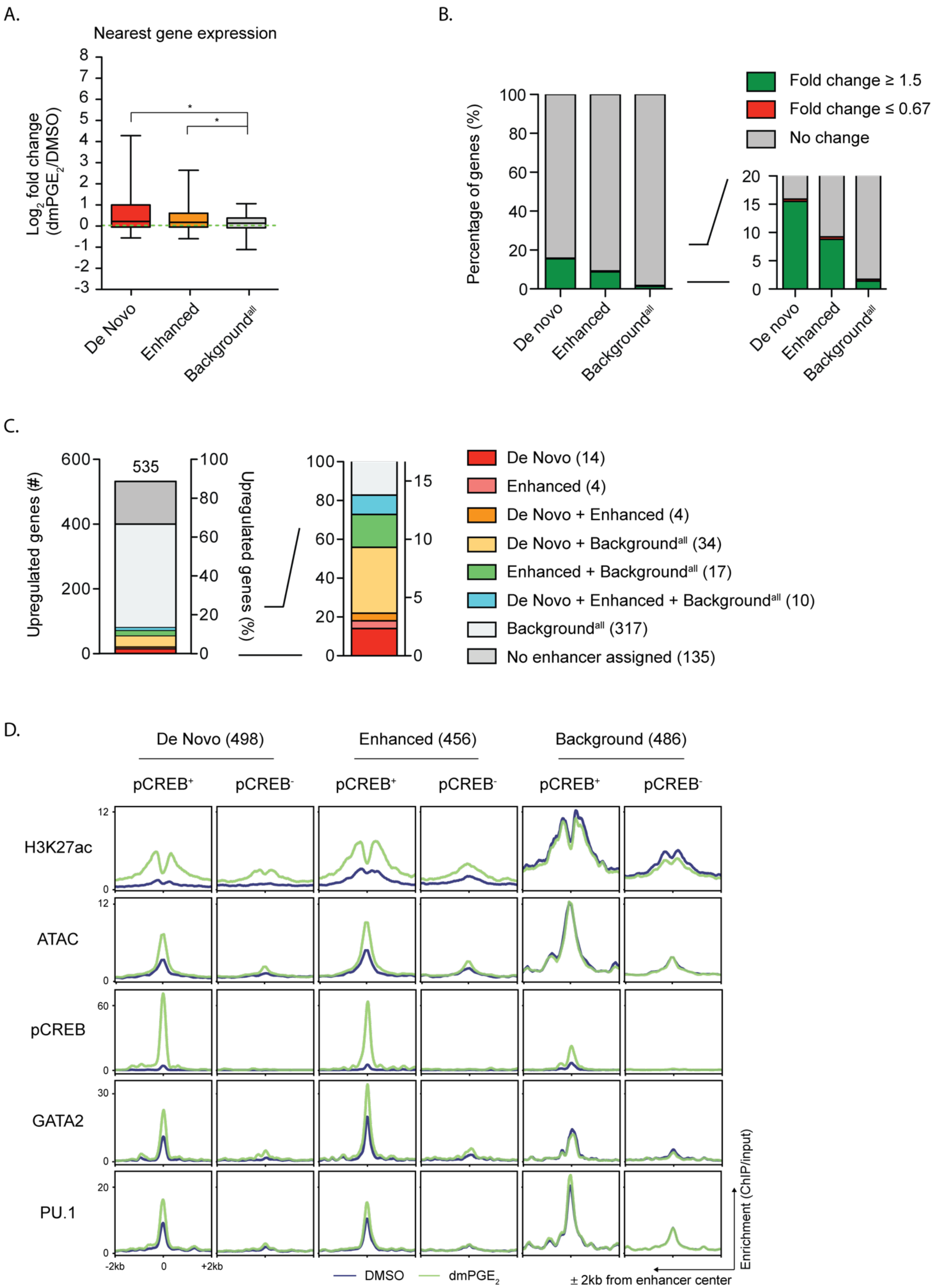

A.
Nearest gene expression
Log2 fold change (dmPGE2/DMSO)
De Novo
Enhanced
Backgroundall
B.
Percentage of genes (%)
Fold change ≥ 1.5
Fold change ≤ 0.67
No change
De novo
Enhanced
Backgroundall
C.
Upregulated genes (#)
Upregulated genes (%)
535
De Novo (14)
Enhanced (4)
De Novo + Enhanced (4)
De Novo + Backgroundall (34)
Enhanced + Backgroundall (17)
De Novo + Enhanced + Backgroundall (10)
Backgroundall (317)
No enhancer assigned (135)
D.
De Novo (498)
Enhanced (456)
Background (486)
pCREB+
pCREB-
H3K27ac
ATAC
pCREB
GATA2
PU.1
-2kb
0
+2kb
DMSO
dmPGE2
± 2kb from enhancer center
Enrichment (ChIP/input)

Supplemental Figure 4. Stimuli-responsive enhancers mediate gene expression changes. (A) Gene expression changes of genes associated with stimuli-responsive and background enhancers. Enhancers were assigned to an individual nearest gene. Only genes with a mapped TSS within 15kb of an enhancer were considered. Box plots shows median, 25th and 75th percentiles, whiskers are from 5th and 95th percentiles. (B) Percentages of enhancer nearest genes with fold changes in expression ≥ 1.5-fold or ″ 0.67-fold for each enhancer category. (C) Upregulated genes with a fold change in expression ≥ 1.5 (535) and their associated enhancers. For all analysis presented in A, B, and C the entire set of background enhancers (25,044) was used. (D) Average enrichment profile of H3K27ac, ATAC accessibility and transcription factors around enhancers before and after $dmPGE_2$ treatment. De Novo, Enhanced or Background enhancers were subset based on the presence or absence of pCREB after $dmPGE_2$. A randomly sampled, comparable number of background enhancers (486) is shown. * = $p < 0.0001$

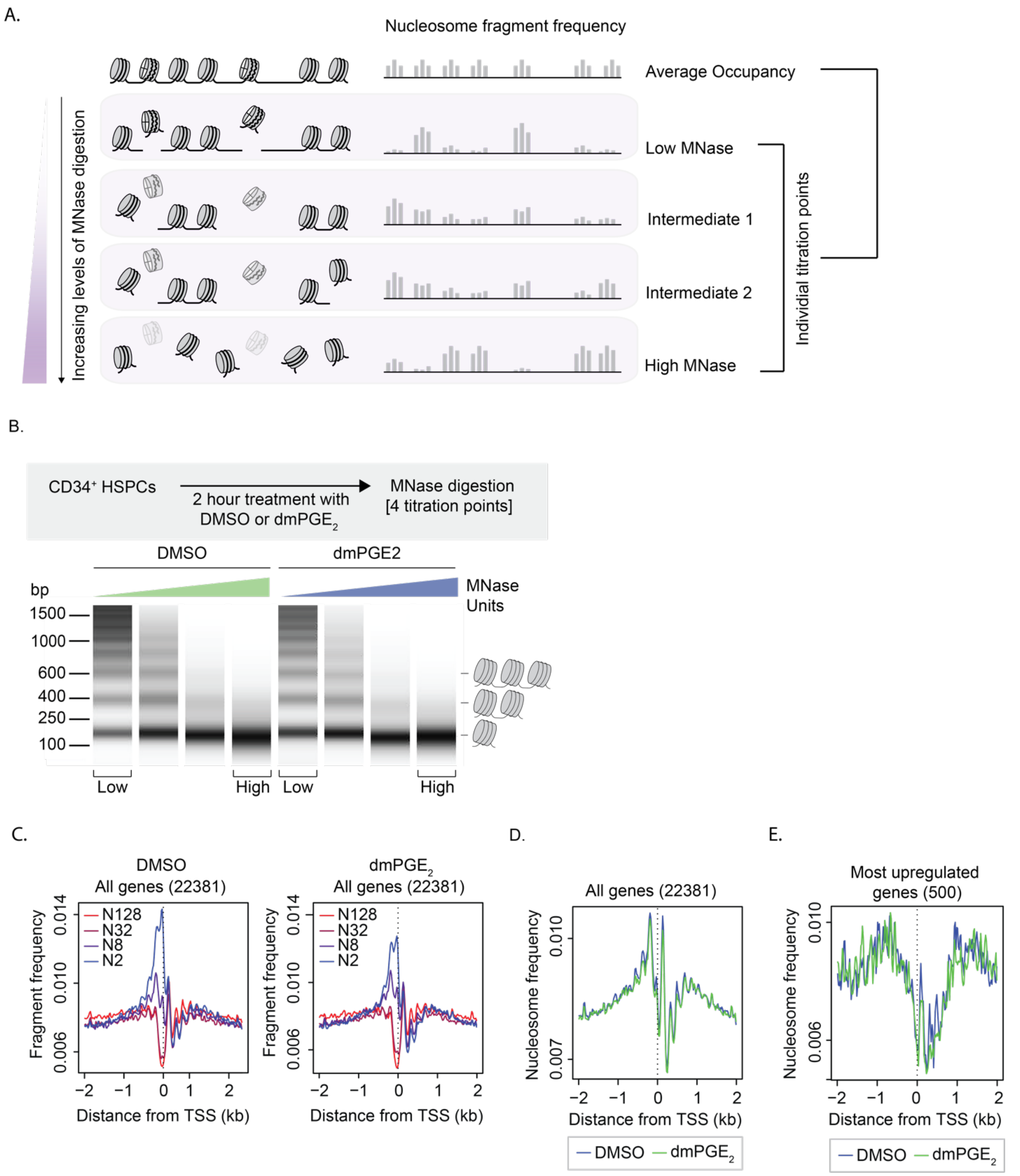
A.
Nucleosome fragment frequency
Average Occupancy
Low MNase
Intermediate 1
Intermediate 2
High MNase
Individial titration points
Increasing levels of MNase digestion
B.
CD34+ HSPCs
2 hour treatment with DMSO or dmPGE2
MNase digestion [4 titration points]
DMSO
dmPGE2
MNase Units
bp
1500
1000
600
400
250
100
Low
High
Low
High
C.
DMSO
All genes (22381)
dmPGE2
All genes (22381)
N128
N32
N8
N2
Fragment frequency
Distance from TSS (kb)
D.
All genes (22381)
Nucleosome frequency
Distance from TSS (kb)
DMSO
dmPGE2
E.
Most upregulated genes (500)
Nucleosome frequency
Distance from TSS (kb)
DMSO
dmPGE2

Supplemental Figure 5. MNase-sequencing in DMSO and dmPGE$_2$ treated HSPCs. (A) Schematic representation of MNase-Seq using 4 titration point. Average nucleosome occupancy is determined through pooled analysis of 4 individual MNase digestion levels, as indicated (n = 3 independent biological experiments). (B) Capillary electrophoresis of digestion products from a typical MNase titration experiment. Cells are stimulated for 2 hours with dmPGE$_2$ or vehicle control (DMSO) after which MNase digestion was performed. (C) MNase-Seq profiles around TSS (transcription start sites) of all genes. Colors indicates MNase concentration levels (2, 8, 32 and 128 Units of MNase), with blue corresponding to the lowest concentration and red corresponding to the highest. (D) The average nucleosome profile at the TSS of all genes in DMSO treated (blue) and dmPGE$_2$ (green) treated HSPCs, as determined from 4 individual MNase titration point per experimental condition. (E) The average nucleosome profile at the TSS of the 500 most upregulated in DMSO treated (blue) and dmPGE$_2$ (green) treated HSPCs, as determined from 4 individual MNase titration point per experimental condition.

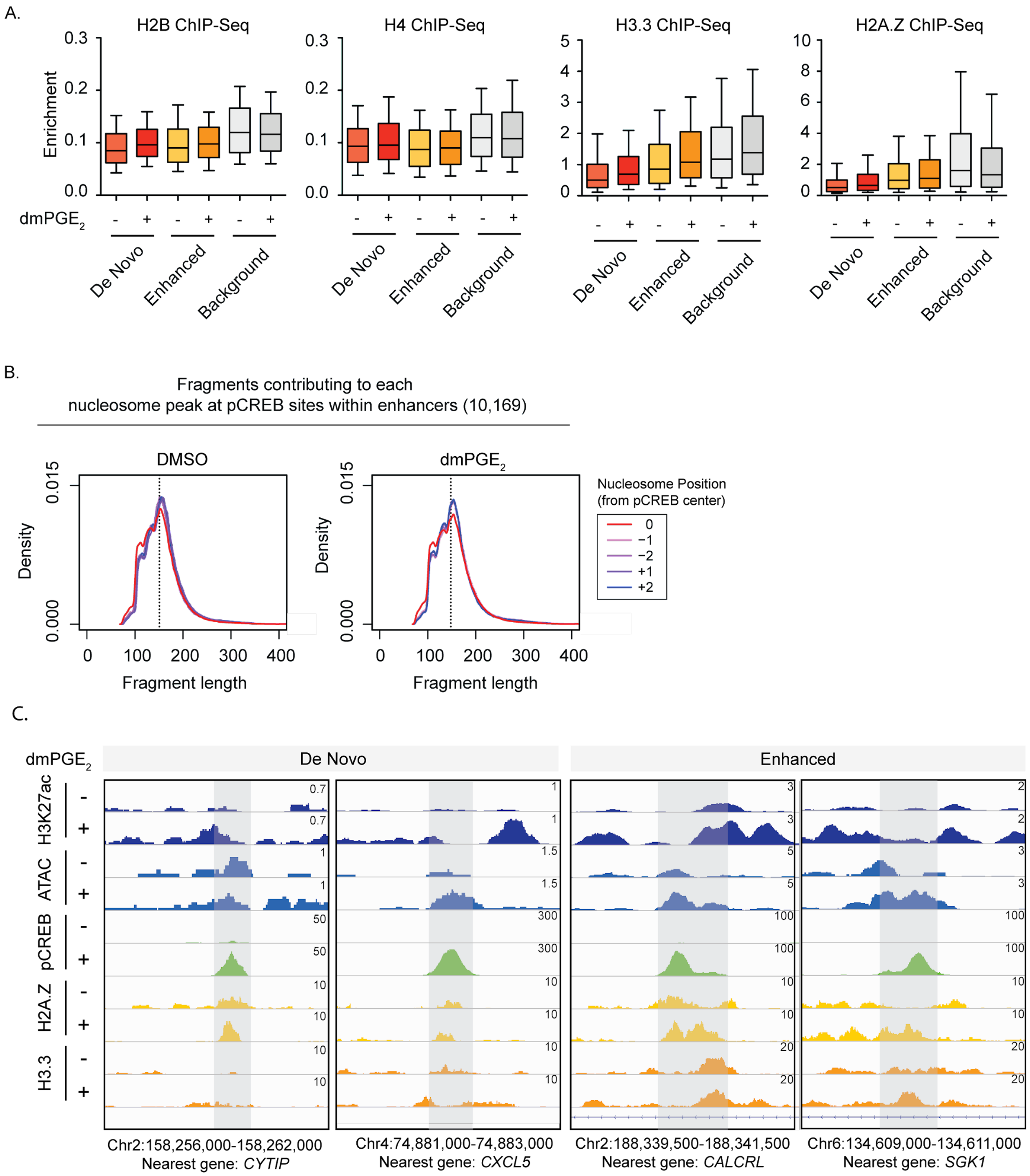
A.
H2B ChIP-Seq
H4 ChIP-Seq
H3.3 ChIP-Seq
H2A.Z ChIP-Seq
Enrichment
dmPGE2
De Novo
Enhanced
Background
B.
Fragments contributing to each nucleosome peak at pCREB sites within enhancers (10,169)
DMSO
dmPGE2
Density
Fragment length
Nucleosome Position (from pCREB center)
0
−1
−2
+1
+2
C.
dmPGE2
De Novo
Enhanced
H3K27ac
ATAC
pCREB
H2A.Z
H3.3
Chr2:158,256,000-158,262,000
Nearest gene: CYTIP
Chr4:74,881,000-74,883,000
Nearest gene: CXCL5
Chr2:188,339,500-188,341,500
Nearest gene: CALCRL
Chr6:134,609,000-134,611,000
Nearest gene: SGK1

Supplemental Figure 6. Histone enrichment and MNase fragment size at enhancers. (A) Box and whisker plots show H2B, H3, H2A.Z and H3.3 ChIP-seq signal enrichment at the central 500bp of stimuli-responsive and background enhancers, in DMSO and $dmPGE_2$ treated HSPCs. Box plots shows median, 25th and 75th percentiles, whiskers are from 10th and 90th percentiles. For analysis presented here, a randomly sampled comparable number of background enhancers (486) is used. (B) Size distribution of DNA fragment reads mapped to the corresponding nucleosome position displayed. Sequencing libraries prepared from MNase-generated fragments were subjected to paired-end sequencing, and the sizes of the fragments were inferred from the positions of the mapped ends. Nucleosome position 0 indicates the nucleosome overlapping with pCREB peak centers. (C) Enrichment of ATAC accessibility, pCREB binding and histone variant deposition in response to $dmPGE_2$ at 4 representative stimuli-response enhancers. Genomic location of presented window and nearest gene are indicated at the bottom of the panel. For all analysis presented in B and D a randomly sampled comparable number of background enhancers (486) is shown.

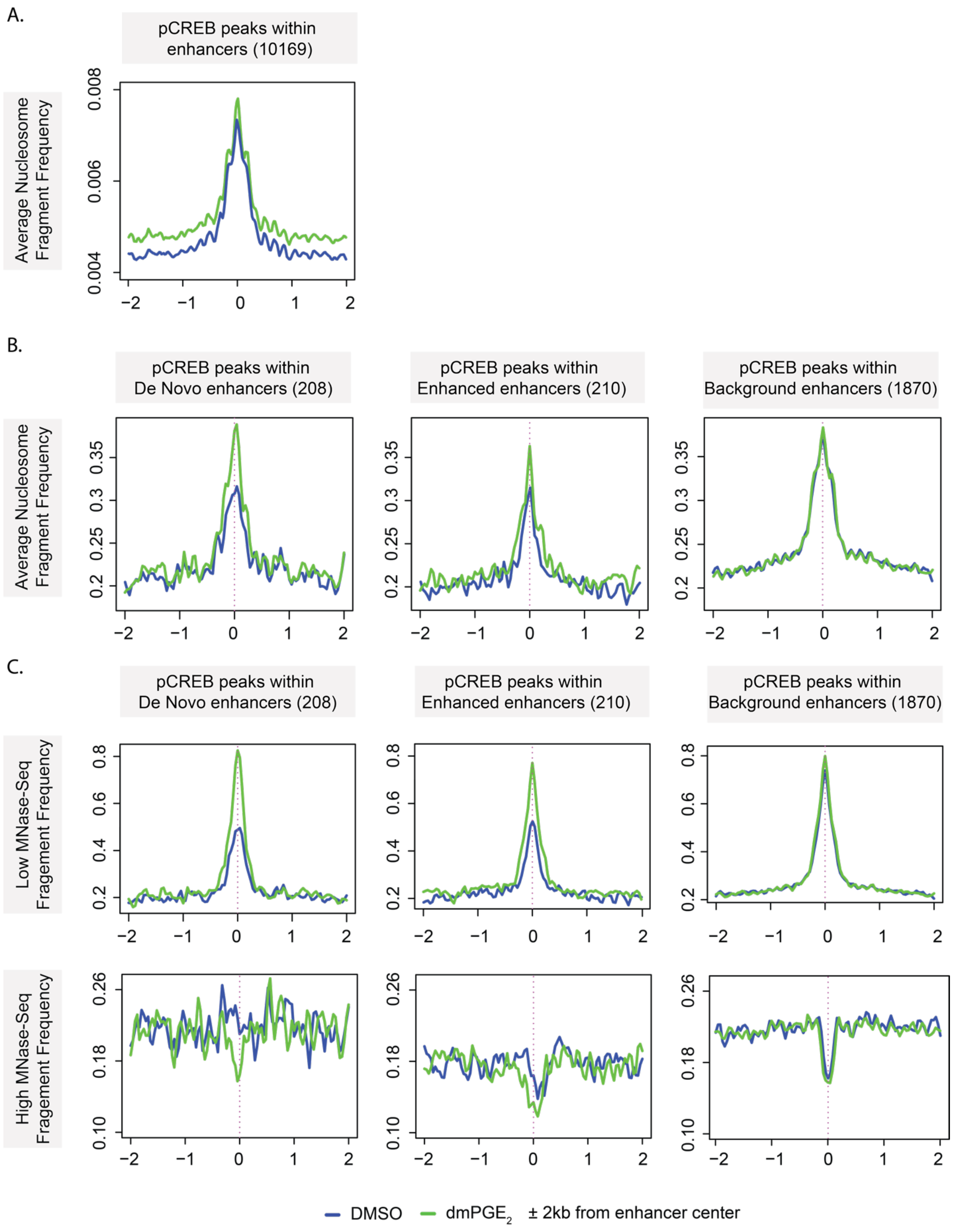
A.
pCREB peaks within enhancers (10169)
Average Nucleosome Fragment Frequency
0.004
0.006
0.008
−2
−1
0
1
2
B.
pCREB peaks within De Novo enhancers (208)
pCREB peaks within Enhanced enhancers (210)
pCREB peaks within Background enhancers (1870)
Average Nucleosome Fragment Frequency
0.2
0.25
0.3
0.35
C.
pCREB peaks within De Novo enhancers (208)
pCREB peaks within Enhanced enhancers (210)
pCREB peaks within Background enhancers (1870)
Low MNase-Seq Fragement Frequency
0.2
0.4
0.6
0.8
High MNase-Seq Fragement Frequency
0.10
0.18
0.26
DMSO
dmPGE2
± 2kb from enhancer center

Supplemental Figure 7. pCREB engages accessible nucleosomes in stimuli-responsive enhancers. (A) Average nucleosome frequency at pCREB peaks located within enhancers before and after dmPGE$_2$ stimulation (n = 3 biologically independent MNase-Seq experiments). (B) Averaged nucleosome occupancy profiles at pCREB-bound sites within stimuli-responsive and background enhancers from 4 MNase titration points (n = 3 biologically independent experiments). (C) Nucleosome profiles of low- and high MNase-Seq at pCREB-bound sites within stimuli-responsive and background enhancers (n = 3 biologically independent experiments). For all analysis presented here a randomly sampled set of background enhancers (486) was used.

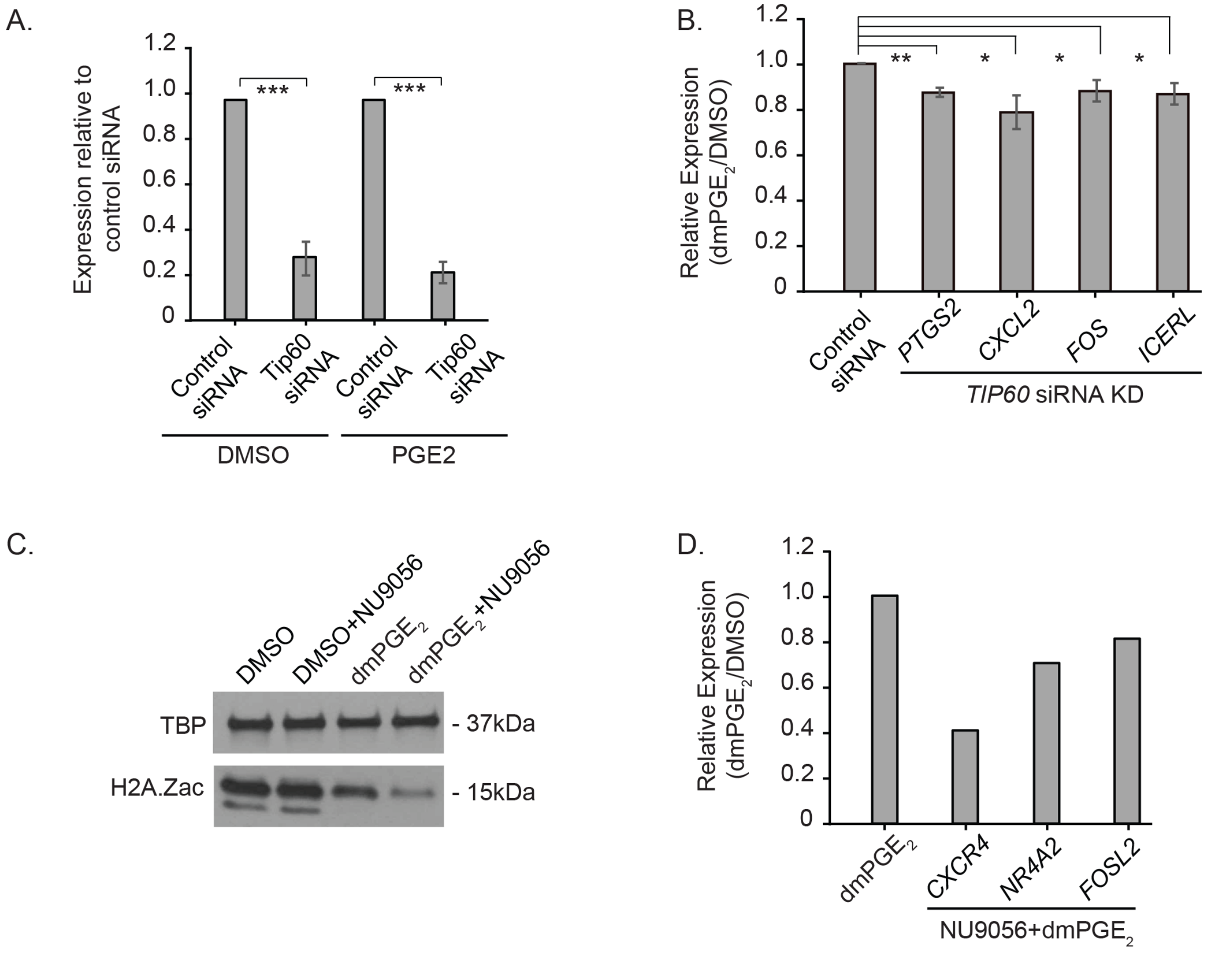
A.
Expression relative to control siRNA
1.2
1.0
0.8
0.6
0.4
0.2
0
***
***
Control siRNA
Tip60 siRNA
Control siRNA
Tip60 siRNA
DMSO
PGE2
B.
Relative Expression (dmPGE2/DMSO)
**
*
*
*
Control siRNA
PTGS2
CXCL2
FOS
ICERL
TIP60 siRNA KD
C.
DMSO
DMSO+NU9056
dmPGE2
dmPGE2+NU9056
TBP
- 37kDa
H2A.Zac
- 15kDa
D.
Relative Expression (dmPGE2/DMSO)
dmPGE2
CXCR4
NR4A2
FOSL2
NU9056+dmPGE2

Supplemental Figure 8. Loss of function of TIP60 affects transcriptional response of dmPGE_2. (A) siRNA-mediated knockdown of *TIP60* in $CD34^+$ HSPCs with DMSO and dmPGE_2 treatment. (B) siRNA-mediated knockdown of *TIP60* compromises dmPGE_2 response on representative target genes, *PTGS2*, *CXCL2*, *FOS*, *ICERL*. Expression of individual genes upon dmPGE2 treatment relative to DMSO is as indicated. (B) Decrease of H2A.Z acetylation under loss of TIP60 function with NU9056 as confirmed by western blot. Experiments were performed with both DMSO and dmPGE_2 treatment. TBP has been used as loading control. (C) NU9056-mediated inhibition of Tip60 function prohibits effective dmPGE_2-induction of representative target genes. Results of one representative experiment out of three are shown. Expression of individual genes upon dmPGE_2 treatment relative to DMSO is as indicated. p-value ≤ 0.005 = ***, p-value ≤ 0.01 = **, p-value ≤ 0.05 = **.

Supplemental materials and methods

Western blotting

Cells were treated for 2 hours, washed in 1X PBS, and collected in RIPA buffer with protease and phosphatase inhibitors. Samples were run on acrylamide gel and transferred onto a nitrocellulose membrane. Membrane was blocked for one hour in 5% of milk or TBS-T and incubated overnight at 4ºC with anti-vinculin (Abcam 73412), anti-TBP (Abcam 818), anti-CREB (Santa Cruz SC-186), anti-pCREB (Ser133, Cell Signaling #9198), anti-H2A.Z (Abcam 4174), or anti-IgG (Cell Signaling #2729). The next day, membranes were washed, incubated with HRP-conjugated secondary antibodies for 1 hour at room temperature, and developed with SuperSignal West Pico Plus Chemiluminescent substrate.

Co-immunoprecipitation (Co-IP)

Co-IP was performed as previously described by Santoriello *et al.,* 2020[1]. Cells were washed twice with 1X PBS. Nuclei were isolated with 0.05% Triton in PBS and lysed in nuclei lysis buffer (20mM Hepes-KOH pH7.9, 25% glycerol, 420mM NaCl, 1.5mM MgCl2, 0.2mM EDTA, 0.5mM DTT). 500ug of protein extracts was used and the salt concentration was diluted from 420mM to 150mM NaCl using 20mM Hepes-KOH pH7.9, 20% glycerol, 0.25mM EDTA, 0.05% NP-40. Cell lysates were pre-cleared with non-antibody bounds beads for 1 hour at 4°C. Antibody bounds protein G Dynabeads were added to pre-cleared lysate and samples incubated overnight at 4°C. Antibodies used: IgG (Cell Signaling #2729), phospho-CREB (Ser133, Cell Signaling #9198). Protein-bead complexes were then washed 5 times with wash buffer (20mM Hepes-KOH pH7.9, 10% Glycerol, 150mM NaCl, 1.5 MgCl2, 0.2mM EDTA, 0.5mM DTT) and beads were boiled in 50µL Laemmli Buffer for 15min at 95°C to elute proteins. Subsequently, samples were subjected to western blot.

qPCR analysis primers

The following PCR primer-pairs were used:

CXCL2 Fwd: AAACCGAAGTCATAGCCACACTC

	Rev: AGCCACCAATAAGCTTCCTCCTTC
CXC4	Fwd: CCTATGCAAGGCAGTCCATGT
	Rev: GGTAGCGGTCCAGACTGATGA
EGR1	Fwd: GCGAGCAGCCCTACGAGCAC
	Rev: TGCAGGCTCCAGGGAAAAGC
FOS	Fwd: TGCCTCTCCTCAATGACCCTGA
	Rev: ATAGGTCCATGTCTGGCACGGA
FOSB	Fwd: TTCTGACTGTCCCTGCCAAT
	Rev: CGGGGTCAGATGCAAAATAC
FOSL2	Fwd: GCAGTTGGGTTTCTGGCTTGAG
	Rev: TCCTGCTACTCCTGGCTCATTC
GAPDH	Fwd: GAAGGTCGGAGTCAACGGATTT
	Rev: GAATTTGCCATGGGTGGAAT
ICER	Fwd: CACCATGGCTGTAACTGGAGATGAC
	Rev: AGGTCCAAGTCAAAGACAGTTACTC
NR4A1	Fwd: GAGTGCACAGAAGAACT
	Rev: CACAGGAGGAGGAAGA
NR4A2	Fwd: CACAGGTTGCAATGCGTTCG
	Rev: TCAATTATTGCTGGCGGTGG
NR4A3	Fwd: CGTCGAAACCGATGTCAGTA
	Rev: GACGACCTCTCCTCCCTTTC
PTGS2	Fwd: GAATCATTCACCAGGCAAATTG
	Rev: TCTGTACTGCGGGTGGAACA
TIP60	Fwd: GACCTACGACATCCTCCAGG
	Rev: CAGGTTCTGGGAATAACTCTTGT

Tip60 Inhibition

For TIP60 knockdown experiments in CD34^{+} HSPCs, siRNA was delivered according to Dharmacon™ Accell™ siRNA delivery protocol for for suspension cells. In brief, lyophilized siRNA was reconstituted in 1x siRNA buffer to 100µM. On day 4 of expansion of CD34^{+} HSPC cells, siRNA was added to 500.000 cells per 1mL of expansion media at

a final concentration of 1μM. Cells were incubated with siRNA and appropriate expansion media for 72 hours or 3 days. On day 7, $CD34^+$ HSPCs were treated with 10μM dmPGE2 or DMSO for 2 hours, as earlier described. Product codes of siRNAs with target transcripts are as follows: A-006301-16-0020 for targeting of human KAT5 (TIP60) and D-001910-10-20 non-targeting pool for negative control.

For inhibitor experiments, U937 cells were incubated with 10μM of the Tip60 inhibitor NU9056 for 24 hours prior to treatment with 10μM dmPGE2 or DMSO for 2 hours.

RNA-Seq

RNA from one million cells was isolated using the RNeasy plus mini kit (Qiagen #74134). 5μg of RNA was subjected to ribosomal and mitochondrial RNA depletion using the RiboZero Gold kit (Human/Mouse/Rat, Epicentre #MRZG12324) according to manufacturer's instructions. The ribo-zero treated RNA wa*s* used to create multiplexed RNA-Seq libraries using the NEBNext Ultra RNA Library Prep Kit (Illumina E7530) according to the manufacturer’s instructions. Briefly 500pg of ribozero treated RNA was fragmented and used to produce cDNA libraries using the NEBnext Ultra RNA library prep kit (NEB, E7530S) according to the manufacturer’s protocol. Purified double-stranded cDNA underwent end-repair and dA-tailing reactions following manufacturer’s reagents and reaction conditions. The obtained DNAs were used for Adaptor Ligation using adaptors and enzymes provided in NEBNext Multiplex Oligos for Illumina (NEB #E7335) and following recommended reaction conditions. Eluted DNA was enriched with PCR reaction using Fusion High-Fidelity PCR Master Mix kit (NEB, M0531S) and specific index primers supplied in NEBNext Multiplex Oligo Kit for Illumina (Index Primer Set 1, NEB, E7335L). Conditions for PCR used are as follows: 98°C, 30 sec; [98°C, 10 sec; 65°C, 30 sec; 72°C, 30 sec] X 15 cycles; 72°C, 5 min; hold at 4°C. PCR reaction mix was purified using Agencourt AMPure XP beads (1X of reaction volume). Libraries were eluted in 20μl elution buffer. All the libraries went through quality control analysis using an Agilent Bioanalyzer and subjected to next-generation sequencing using Illumina Hiseq 2500 platform. Quality control of RNA-Seq datasets was performed by FastQC and Cutadapt to remove adaptor sequences and low-quality regions. The high-quality reads were aligned to UCSC hg19 for human using Tophat 2.0.11 without novel splicing form calls.

Transcript abundance and differential expression were calculated with Cufflinks 2.2.1. FPKM values were used to normalize and quantify each transcript.

ChIP-Seq

For ChIP-Seq experiments the following antibodies were used: H3K27ac (Abcam ab4729), H3K4me1 (Abcam ab8895), H3K27me3, (Abcam ab195477), pCREB (Ser133, Cell Signlling #9198), CREB (Santa Cruz sc186X), GATA2 (Santa Cruz sc9008X), H2A.Z (Abcam ab4174), H2A.Zac (Abcam ab18262), H2B (Abcam ab1790), H3.3 (Milipore #09-838), H4 (Abcam ab7311), p300 (Millipore #05-257), and Tip60 (generous gift from Bruno Amati). ChIP experiments were performed as previously described by Trompouki *et al.,* 2011[2]. Briefly, 20 million cells were crosslinked by the addition of 1/10 volume 11% fresh formaldehyde for 10 min at room temperature. The crosslinking was quenched by the addition of 1/20 volume 2.5M glycine for 5 minutes. Cells were washed twice with ice-cold PBS. Cells were lysed in 10mL of Lysis buffer 1 (50mM HEPES-KOH, pH 7.5, 140mM NaCl, 1mM EDTA, 10% glycerol, 0.5% NP-40, 0.25% Triton X-100, plus protease and phosphatase inhibitors) for 10 min at 4°C. After centrifugation, cells were resuspended in 10 mL of Lysis buffer 2 (10mM Tris-HCl, pH 8.0, 200mM NaCl, 1mM EDTA, 0.5mM EGTA, plus protease and phosphatase inhibitors) for 10 min at room temperature. Cells were pelleted and resuspended in 3mL of sonication buffer (10mM Tris-HCl, pH 8.0, 100mM NaCl, 1mM EDTA, 0.5mM EGTA, 0.1% Na-Deoxycholate, 0.05% N-lauroylsarcosine, plus protease and phosphatase Inhibitors) and sonicated in a Bioruptor sonicator for 36 cycles of 30 sec each followed by a 1min resting interval. Samples were centrifuged for 10min at 18,000g and 1% Triton-X was added to the supernatant. Prior to the immunoprecipitation, 50mL of protein G beads (Invitrogen 100-04D) for each reaction were washed twice with PBS, 0.5% BSA twice. Finally, the beads were resuspended in 250μL of PBS, 0.5% BSA and 5μg of each antibody. Beads were rotated for at least 6 hours at 4°C and then washed twice with PBS with 0.5% BSA. Cell lysates were added to the beads and incubated at 4°C overnight. Beads were washed 1x with (20mM Tris-HCl. pH 8, 150mM NaCl, 2mM EDTA, 0.1% SDS, 1% Triton X-100), 1x with (20mM Tris-HCl, pH 8, 500mM NaCl, 2mM EDTA, 0.1% SDS, 1% Triton X-100), 1x with (10mM Tris-HCl, pH 8, 250nM LiCl, 2mM EDTA, 1% NP4-0) and 1x with TE and finally resuspended

in 200μL elution buffer (50mM Tris-Hcl, pH 8.0, 10mM EDTA, 0.5%–1% SDS). 50μL of cell lysates prior to addition to the beads was kept as input. Crosslinking was reversed by incubating samples at 65°C for at least 6 hours. Afterwards the cells were treated with RNase and proteinase K and the DNA was extracted by Phenol/Chloroform extraction.

ChIP-Seq libraries were prepared using the following protocol. End repair of immunoprecipitated DNA was performed using the End-It End-Repair kit (Epicentre, ER81050) and incubating the samples at 25°C for 45 min. End-repaired DNA was purified using AMPure XP Beads (1.8X of the reaction volume) (Agencourt AMPure XP – PCR purification Beads, BeckmanCoulter, A63881) and separating beads using DynaMag-96 Side Skirted Magnet (Life Technologies, 12027). A-tails were added to the end-repaired DNA using NEB Klenow Fragment Enzyme (3'-5' exo, M0212L), 1X NEB buffer 2 and 0.2mM dATP (Invitrogen, 18252-015) and by incubating the reaction mix at 37μC for 30 min. A-tailed DNA was cleaned up using AMPure beads (1.8X of reaction volume). Subsequently, cleaned up A-tailed DNA went through Adaptor ligation reaction using Quick Ligation Kit (NEB, M2200L) following manufacturer's protocol. Adaptor-ligated DNA was first cleaned up using AMPure beads (1.8X of reaction volume), eluted in 100μl and then size-selected using AMPure beads (0.9X of the final supernatant volume, 90μl). Adaptor-ligated DNA fragments of proper size were enriched with PCR reaction using Fusion High-Fidelity PCR Master Mix kit (NEB, M0531S) and specific index primers supplied in NEBNext Multiplex Oligo Kit for Illumina (Index Primer Set 1, NEB, E7335L). Conditions for PCR used are as follows: 98°C, 30 sec; [98°C, 10 sec; 65°C, 30 sec; 72°C, 30 sec] X 15 to 18 cycles; 72°C, 5 min; hold at 4°C. PCR enriched fragments were cleaned up using AMPure beads (1X of reaction volume). Libraries were eluted in 20μl elution buffer. All the libraries went through quality control analysis using an Agilent Bioanalyzer and subjected to next-generation sequencing using Illumina Hiseq 2500 platform. All the libraries went through quality control analysis using an Agilent Bioanalyzer and subjected to next-generation sequencing using Illumina Hiseq 2500 platform. All ChIP-Seq datasets were aligned to UCSC build version hg19 of the human genome using Bowtie2 (version 2.2.1; Langmead *et al.,* 2012[3]) with the following parameters: -end-to-end, -N0, -L20. We used the MACS2 version 2.1.0 (Zhang *et al.,* 2008[4]) peak-finding algorithm to identify regions of ChIP-Seq peaks, with a q-value threshold of enrichment of 0.05 and false

discovery rate of < 0.01 for all datasets. The genome-wide occupancy profile figures were generated by deeptools (Ramirez *et al.,* 2016[5]) using the reference-point mode and the scale-regions mode. The genomic distribution of peaks was plotted using the ChIPSeeker R package, annotatePeak to assign peaks to a genomic annotation, which includes whether a peak is in the TSS, Exon, 5' UTR, 3' UTR, Intronic or Intergenic. The genome annotation is from the R-bioconductor annotation packages. Heat maps of the ChIP-Seq binding were generated using the input normalized results of the MACS peak calling output. The outputted bedGraph files were converted to BigWig files. Those files were then processed using computeMatrix and plotHeatmap tools within the deeptools 3.0 package. Enhancers were assigned to genes using GREAT to the nearest genes within 15kb of peaks.

Defining Enhancer Categories

We used the SPP package to call clusters of H3K27ac enrichment, normalized to input, from ChIP-Seq data (Kharchenko *et al.,* 2008[6]). Regions within 500bp of each other were merged and only regions reproduced between two independent biological H3K27ac ChIP-Seq replicates (in either sample dmPGE$_2$ or DMSO) were included for further analysis. Enhancers were defined as TSS distal H3K27ac regions that are ≥ 1kb in length and located ≥ 2kb away from TSS. This yielded a total of 25,998 H3K27ac enriched ChIP-Seq regions, here named enhancers. P-value was computed using paired t-test between dmPGE$_2$ and DMSO on enrichment values for every region.

Called enhancers were classified based on the three following criteria and using cut-off described in the table below: (1) H3K27ac enrichment in each replicate in each condition, (2) delta H3K27ac enrichment upon stimulation, (3) p-value of ⊗ H3K27ac enrichment. All regions not classified as not meeting above mentioned criteria were classified as 'Background' enhancers.

	H3K27ac Enrichment		⊗ Enrichment	Significance
	DMSO	dmPGE$_2$	[dmPGE$_2$-DMSO]	p-value
De Novo	″ 1	≥ 2	≥ 1	″ 0.05

Enhanced	≥ 1	≥ 3	≥ 2	″ 0.05

ATAC-Seq

50,000 cells per condition were harvested by spinning at 500g for 5 min, 4°C. Cells were washed once with 50µl of cold 1X PBS and spun down at 500g for 5 min, 4°C. After discarding supernatant, cells were lysed using 50µl cold lysis buffer (10mM Tris-HCl pH 7.4, 10mM NaCl, 3 mM $MgCl_2$, 0.1% IGEPAL) and spun down immediately at 500g for 10 min at 4°C. Then the cells were precipitated and kept on ice and subsequently resuspended in 25µl 2X Tagment DNA Buffer (Illumina Nextera kit), 2.5µl Transposase enzyme (Illumina Nextera kit, 15028252) and 22.5µl Nuclease-free water in a total of 50uL reaction for 1 hour at 37°C. DNA was then purified using Qiagen MinElute PCR purification kit (28004) in a final volume of 10µl. Libraries were constructed according to Illumina protocol using the DNA treated with transposase, NEB PCR master mix, Sybr green, universal and library-specific Nextera index primers. The first round of PCR was performed under the following conditions: 72°C, 5 min; 98°C, 30 sec; [98°C, 10 sec; 63°C, 30 sec; 72°C, 1 min] X 5 cycles; hold at 4°C. Reactions were kept on ice and using a 5µL reaction aliquot, the appropriate number of additional cycles required for further amplification was determined in a side qPCR reaction: 98°C , 30 sec; [98°C, 10 sec; 63°C, 30 sec; 72°C, 1 min] X 20 cycles; hold at 4°C. Upon determining the additional number of PCR cycles required further for each sample, library amplification was conducted using the following conditions: 98°C, 30 sec; [98°C, 10 sec; 63°C, 30 sec; 72°C, 1 min] X appropriate number of cycles; hold at 4°C. Libraries prepared went through quality control analysis using an Agilent Bioanalyzer and then subjected to next generation sequencing using Illumina Hiseq 2500 platform. We used the MACS2 version 2.1.0 (Zhang *et al.,* 2008[4]) peak-finding algorithm to identify regions of ATAC-Seq peaks, with the following parameter -- nomodel --shift -100 --extsize 200. A q-value threshold of enrichment of 0.05 was used for all datasets.

MNase-Seq

$CD34^+$ HSPC cells were crosslinked by the addition of 1/10 volume 11% fresh formaldehyde for 10 min at room temperature. The crosslinking was quenched by the

addition of 1/20 volume 2.5M glycine for 5 minutes. Cells were washed twice with ice-cold PBS. For MNase digestion, the nuclei pellet was resuspended in MNase digestion buffer (50mM Tris, pH 7.4, 25mM KCl, 4mM MgCl2, 1mM CaCl2, 12.5% Glycerol and COMPLETE protease inhibitors (Roche)). Digestion took place with 10^6 cells per titration point in a volume of 500µl MNase digestion buffer. Either 1, 2, 4, 8, 16, 32, 64, 128 or 256 units of MNase (Worthington Biochemical) were added to pre-warmed nuclei and incubated at 25 °C for 15 min. Digestion was halted with 25mM EDTA/EGTA and 0.5% SDS and 125mM NaCl was added to the samples. Digestions were incubated with RNase (Roche) for 1 hour at 37 °C, with proteinase K (Roche) for 1 hour at 55 °C, and cross-link reversal was performed at 65 °C for 16 hours. DNA was purified by Phenol/Chloroform extraction and ethanol precipitation. MNase digestion was evaluated on a 2% agarose gel and fragments from four MNase concentrations representing 10%, 25%, 50% and 75% mono-nucleosomal fragments were individually prepared for next generation sequencing. Ampure SPRI beads (Beckman Coulter) were used in a double size selection with ratios of 0.7X and 1.7X to obtain a range of fragment sizes from ∼100 to 1,000bp. DNA was eluted from the beads and used as input into the library preparation protocol. DNA libraries were prepared for each individual titration point using the NEBNext Ultra II DNA Library Prep Kit for Illumina (E7370, New England Biolabs) and barcoded using NEBNext Multiplex Oligos for Illumina (Index Primers Set 1 & 2; New England Biolabs). Number of PCR cycles was calculated using a real-time qPCR-based approach (Lion *et al.,* 2020[7]). Libraries prepared went through quality control analysis using an Agilent Bioanalyzer. Four barcoded titration libraries were pooled in one sample, and paired-end sequencing on an Illumina Hiseq 2500 instrument was performed. Three biological replicated were sequenced. The sequenced paired-end reads were mapped to hg19 using Bowtie aligner v. 0.12.9. Only uniquely mapped reads with no more than two mismatches were retained. The reads with the insert sizes <50 bp or >500 bp were filtered out. Genomic positions with the numbers of mapped tags above the significance threshold of *Z*-score=7 were identified as anomalous, and the tags mapped to such positions were discarded. Read frequencies were computed in 300bp non-overlapping bins for each titration point independently. The read frequencies were normalized by the corresponding

library sizes to represent values per one million of mapped reads. Nucleosome occupancy analysis was carried out as previously described by Mieczkowski *et al.,* 2016[8].

Supplemental references

1 Santoriello, C. *et al.* RNA helicase DDX21 mediates nucleotide stress responses in neural crest and melanoma cells. *Nat Cell Biol* **22**, 372-379, doi:10.1038/s41556-020-0493-0 (2020).

2 Trompouki, E. *et al.* Lineage regulators direct BMP and Wnt pathways to cell-specific programs during differentiation and regeneration. *Cell* **147**, 577-589, doi:10.1016/j.cell.2011.09.044 (2011).

3 Langmead, B. & Salzberg, S. L. Fast gapped-read alignment with Bowtie 2. *Nat Methods* **9**, 357-359, doi:10.1038/nmeth.1923 (2012).

4 Zhang, Y. *et al.* Model-based analysis of ChIP-Seq (MACS). *Genome Biol* **9**, R137, doi:10.1186/gb-2008-9-9-r137 (2008).

5 Ramirez, F. *et al.* deepTools2: a next generation web server for deep-sequencing data analysis. *Nucleic Acids Res* **44**, W160-165, doi:10.1093/nar/gkw257 (2016).

6 Kharchenko, P. V., Tolstorukov, M. Y. & Park, P. J. Design and analysis of ChIP-seq experiments for DNA-binding proteins. *Nat Biotechnol* **26**, 1351-1359, doi:10.1038/nbt.1508 (2008).

7 Lion, M., Muhire, B., Namiki, Y., Tolstorukov, M. Y. & Oettinger, M. A. Alterations in chromatin at antigen receptor loci define lineage progression during B lymphopoiesis. *Proc Natl Acad Sci U S A* **117**, 5453-5462, doi:10.1073/pnas.1914923117 (2020).

8 Mieczkowski, J. *et al.* MNase titration reveals differences between nucleosome occupancy and chromatin accessibility. *Nat Commun* **7**, 11485, doi:10.1038/ncomms11485 (2016).
